# The Prognostic Value of Nutritional and Immune Indices for Stage IB Non‐Small Cell Lung Cancer Patients: Insights From a Retrospective Cohort Study

**DOI:** 10.1002/cam4.71089

**Published:** 2025-07-28

**Authors:** Jianqi Hao, Song He, Cong Chen, Wenying Xu, Xiaohu Hao, Nanzhi Luo, Chenglin Guo, Qiang Pu, Lunxu Liu

**Affiliations:** ^1^ Department of Thoracic Surgery and Institute of Thoracic Oncology, West China Hospital Sichuan University Chengdu Sichuan China; ^2^ Frontiers Science Center for Disease‐Related Molecular Network, West China Hospital Sichuan University Chengdu Sichuan China; ^3^ Department of Nursing, West China Hospital Sichuan University/West China School of Nursing, Sichuan University Chengdu Sichuan China; ^4^ Western China Collaborative Innovation Center for Early Diagnosis and Multidisciplinary Therapy of Lung Cancer Sichuan University Chengdu China

**Keywords:** nutritional and immune indices, postoperative recurrence, predictive model, stage IB non‐small cell lung cancer

## Abstract

**Background:**

Postoperative recurrence is a critical factor affecting the prognosis of stage IB non‐small cell lung cancer (NSCLC). We aimed to evaluate the prognostic value of Nutritional and Immune Indices (NII) for postoperative recurrence among IB NSCLC patients.

**Methods:**

Clinical data of patients with pathological stage IB NSCLC who underwent surgical resection were collected. Cut‐off values for NII were calculated using ROC curves, and patients were grouped accordingly. Univariable and multivariable logistic regression analyses were conducted to identify the association between NII and prognosis. A predictive model and corresponding nomogram were developed based on the identified risk factors. The model's performance was evaluated using the AUC and C‐index. Additionally, interactions between NII and tumor marker indices were assessed, and recurrence‐free survival (RFS) was analyzed using the Cox proportional hazards model.

**Results:**

A total of 918 patients were included in this study. The results showed that patients with higher preoperative PNI or lower SII, PLR, and MLR had better RFS. For postoperative NII, patients with a higher postoperative PNI or lower PLR demonstrated better RFS. Preoperative PNI (HR = 0.920 [0.910, 0.940], *p* < 0.0001), postoperative MLR (HR = 1.428 [1.015, 2.010], *p* = 0.041) and △ (post‐pre) PNI (HR = 1.018 [1.002, 1.034], *p* = 0.031) were identified as independent prognostic factors for postoperative recurrence in stage IB NSCLC. The nomogram model indicated that preoperative PNI was the optimal predictor for postoperative recurrence, achieving the highest C‐index (0.658). Additionally, the interaction between preoperative PNI and NSE emerged as an independent prognostic factor for RFS in stage IB NSCLC patients, with a HR of 1.176 (95% CI: 1.159–1.200, *p* = 0.004).

**Conclusions:**

This study underscores the value of an NII‐based prognostic strategy, with preoperative PNI emerging as a particularly reliable indicator of postoperative recurrence risk in stage IB NSCLC patients.

**Abbreviations**
CEA: carcinoembryonic antigen
CI: confidence intervals
MLR: monocyte‐to‐lymphocyte ratio
NII: nutritional and immune indices
NLR: neutrophil‐to‐lymphocyte ratio
NSCLC: non‐small cell lung cancer
NSE: neuron‐specific enolase
OS: overall survival
PLR: platelet‐to‐lymphocyte ratio
PNI: prognostic nutritional index
RFS: recurrence‐free survival
ROC: receiver operating characteristic
SII: systemic immune‐inflammation index
TM: tumor marker

## 1 Introduction

Lung cancer is a highly fatal malignancy globally, with non‐small cell lung cancer (NSCLC) accounting for approximately 85% of all lung cancer cases [1]. Approximately 20%–30% of NSCLC patients are diagnosed with stage IB [2, 3]. Surgery is one of the primary treatment options for stage IB NSCLC [4]. Stage IB NSCLC is categorized into three main subtypes based on tumor size, lymph node involvement, distant metastasis, and pleural invasion: T2aN0M0, T2CentrN0M0, T2ViscPIN0M0, according to the TNM classification guidelines [5]. Due to the biological heterogeneity of different tumor subtypes and the lack of standardized postoperative adjuvant therapeutic regimens, the prognosis for IB stage NSCLC patients varies significantly, with an overall 5‐year survival rate of 58%. Recurrence or metastasis (approximately 30%–70%) is a major cause of poor prognosis in stage IB NSCLC patients [6, 7, 8], most of which may occur within 2–3 years after surgery [9, 10]. Therefore, apart from selecting appropriate adjuvant therapy strategies, continuous postoperative follow‐up to monitor tumor recurrence and adjust treatment strategies is equally crucial for improving the prognosis of stage IB NSCLC patients.

Recent studies have demonstrated that the individual's immune and nutritional status can influence the tumor microenvironment, as well as the proliferation and dissemination of cancer cells, by modulating systemic inflammatory responses and antitumor immunity; consequently, this may have an impact on tumor progression, recurrence, efficacy of anticancer therapies, and prognosis of cancer patients [11, 12]. Following the basic research, researchers have identified and evaluated a series of blood nutritional and immunological composite indices, including prognostic nutritional index (PNI), systemic immune‐inflammation index (SII), neutrophil‐to‐lymphocyte ratio (NLR), platelet‐to‐lymphocyte ratio (PLR), and monocyte‐to‐lymphocyte ratio (MLR) which have been demonstrated to effectively predict the prognosis of patients with various types of tumors [13, 14, 15, 16, 17]. Since these indices are derived from routine and easily accessible blood test data, prognostic assessment strategies based on these nutritional or immunological markers are expected to become important and reliable tools for evaluating post‐treatment survival and recurrence in cancer patients. However, the prognostic efficacy of different nutritional and immune indices varies for a certain type of cancer, and the immune and nutritional status of tumor patients could fluctuate during disease progression and treatment. Therefore, it is a topic worthy of further exploration to determine an appropriate prognosis assessment strategy by utilizing the nutritional and immunological indices for post‐treatment survival outcomes.

Therefore, the current retrospective study aims to analyze the correlations between composite perioperative (preoperative, postoperative, delta between preoperative and postoperative) nutritional and immunological indices (including PNI, SII, NLR, PLR, and MLR) and postoperative recurrence and survival among stage IB NSCLC patients. Based on these findings, the study seeks to propose a Nutritional and Immune Indices (NII)‐based prognostic assessment strategy, which could serve as a reliable reference for guiding postoperative follow‐up and personalized adjuvant therapy decision making, ultimately improving long‐term survival rates and quality of life for patients.

## 2 Material and Methods

This study received approval from the Institutional Review Board of West China Hospital (No. 2025639). This research used patient data with written consent from the patient to use his or her medical records for comprehensive research purposes. This project was registered in the Chinese Clinical Trial Registry. We present the following article in accordance with the STROCSS Guidelines [18].

## 3 Patients

We retrospectively examined data from consecutive patients with stage IB NSCLC who underwent radical resection of lung cancer at the Department of Thoracic Surgery of West China Hospital from September 2005 to December 2014. The TNM stage of each patient was reclassified after surgery based on the pathology results and the recently revised Ninth Edition Lung Cancer Stage Classification. Patient demographics, hematological parameters, tumor characteristics, preoperative treatment, and follow‐up results were reviewed in detail from the electronic medical record system and the follow‐up system. The inclusion criteria were as follows: (a) histologically diagnosed; (b) stage IB according to the 9th edition of TNM staging; (c) primary lung cancer. The exclusion criteria were (a) history of other cancer; (b) uncompleted blood nutritional and immune parameters; (c) lack of follow‐up data, including RFS, OS.

## 4 The Definition of Nutritional and Immune Indices (NII)

The Nutritional and Immune Indices (NII) was defined as a prognostic index based on immunological and nutritional parameters obtained from blood tests, incorporating three dimensions: preoperative, postoperative, and post‐to‐preoperative differences (Δ) in PNI (prognostic nutritional index), SII (systemic immune‐inflammation index), PLR (platelet‐to‐lymphocyte ratio), NLR (neutrophil‐to‐lymphocyte ratio) and MLR (monocyte‐to‐lymphocyte ratio). The NII‐based prognostic assessment strategy also integrates interactions between NII indices and tumor markers to provide a more comprehensive prediction of postoperative recurrence and overall survival in stage IB NSCLC patients. The latest laboratory data within 1 month before and after the operation were used for the NII calculation. The PNI is 10 × serum albumin (g/dL) + 5 × total lymphocyte count (/nL). The SII is platelet × neutrophil/lymphocyte. The PLR is platelet‐lymphocyte ratio. The NLR is neutrophil‐lymphocyte ratio. The MLR is monocyte‐lymphocyte ratio.

## 5 Variables and Outcomes

The collected clinical information of patients includes age, sex, preoperative and postoperative hematological indicators, including serum albumin, total peripheral blood lymphocyte count, platelet count, neutrophil count, monocyte count, and total lymphocyte count. The primary outcome measures were patients' 0.5‐, 1‐, 1.5‐, and 2‐year recurrence disease‐free survival (RFS) and overall survival (OS).

## 6 Statistical Analysis

Statistical analyses were performed using the R (version 4.0.3). Data from two or more groups were compared using the unpaired two‐tailed Student's *t*‐test and one‐way ANOVA. The significance of patients' NIIs indicators between different subgroups was analyzed using the chi‐squared test. Both univariate and multivariate analyses were performed using the SPSS Statistics (version 26.0). Continuous variables are expressed as the median (interquartile range) or mean ± standard deviation, and categorical variables are presented as the frequency (percentage). *p* < 0.05 was considered statistically significant difference. The Variance Inflation Factor (VIF) was calculated to assess the degree of multicollinearity among the variables. The cutoff values for each index were established through ROC (Receiver Operating Characteristic) curve analysis. ROC plots were analyzed using the “pROC” package in R and visualized with the “ggplot2” package to predict the sensitivity of each effector in NSCLC patients at various disease stages. Concurrently, KM (Kaplan–Meier) plots were analyzed statistically and visualized using the “survival” and “survminer” packages in R to predict the survival of NSCLC patients at different stages for each effector. The nomogram was utilized to identify the optimal predictive model. Nomogram plots were constructed using the “rms” and “survival” R packages. These models enable the accurate prediction of the survival of NSCLC patients based on the scores of individual risk factors and their combined total. Additionally, the RFS and OS of patients were predicted separately. In addition, calibration curves and the concordance index (C‐index) were used to show the accuracy and reliability of the survival predictions. An interaction term analysis was conducted by employing blood nutritional indices and tumor markers as variables. The interaction between NII and tumor markers was obtained by multiplying the optimal predictive indices of NII with preoperative and postoperative tumor markers. Subsequently, multivariate Cox regression analyses were performed based on these interaction terms. Chi‐square tests and independent samples *t*‐tests were used to assess whether the subgroup of patients with tumor marker data is representative of the overall cohort. All datasets used for analysis in this study are available upon request by contacting the corresponding author.

## 7 Results

**FIGURE 1:**
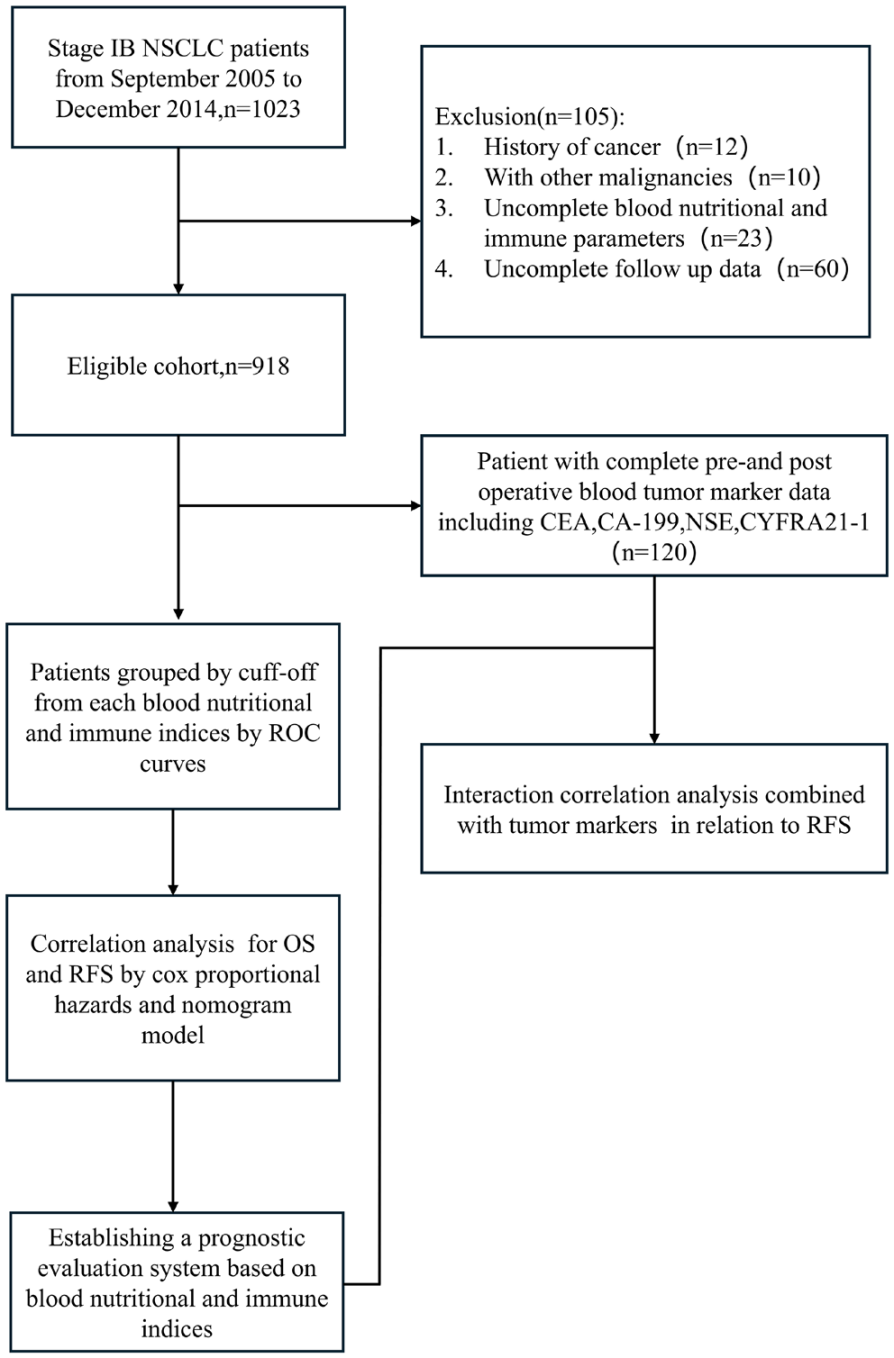
Flowchart of patient selection.

Among the initially screened 1023 stage IB NSCLC patients treated via thoracoscopic surgery, 22 patients with a history of other tumors, 23 patients missing hematological examination results, and 60 patients with incomplete postoperative follow‐up records were excluded. Ultimately, 918 patients meeting the inclusion and exclusion criteria were selected for subsequent analysis. Among these patients, 120 had complete preoperative and postoperative tumor marker examination results (Figure 1). The information of tumor markers is shown in Table 7. The baseline characteristics of these 120 patients are shown in Table 8. Among the 918 included stage IB NSCLC patients, 528 (57.52%) were male and 390 (42.28%) were female, with a median age of 59 years (range: 28–86). Additionally, 516 patients (56.21%) had a history of smoking. Furthermore, 410 patients (44.66%) received standard postoperative adjuvant chemotherapy. The median tumor size was 2.9 cm, with 150 (16.34%) of patients presenting with pleural invasion. Most patients underwent VATS surgery, while a smaller group received traditional open thoracotomy. Postoperative pathology revealed that 670 (72.98%) had adenocarcinoma and 248 (27.02%) had squamous cell carcinoma. Regarding tumor differentiation, 442 (48.15%) patients were classified as poorly differentiated, while 476 (51.85%) patients were classified as moderately to well differentiated (Table 1). The median follow‐up period was 24.8 months.

**TABLE 1:** Clinicopathologic features of patients involved in the study (*n* = 918).

Variables	Type	Value	Subtype	Number
Age	Mean ± SD	59.57 ± 10.11		
	Min–Max	28–86		
Gender			Male	528 (57.52%)
			Female	390 (42.28%)
BMI	Mean ± SD	22.95 ± 3.13		
	Min–Max	14.34–36.20		
Complication			No	467 (50.87%)
			Yes	451 (49.13%)
Diameter	Mean ± SD	2.96–1.11		
	Min–Max	0.30–5.40		
Pathological type			Adenocarcinoma	670 (72.98%)
			Squamous	248 (27.02%)
Differentiate			Low	442 (48.15%)
			Medium‐High	476 (51.85%)
Pleural invasion			Yes	594 (64.71%)
			No	83 (9.04%)
			NA	241 (26.25%)
Site			Upper‐left	231 (25.16%)
			Upper‐right	302 (32.90%)
			Lower‐left	231 (25.16%)
			Lower‐right	170 (18.52%)
			Centre‐right	65 (7.08%)
Smoking history			No	516 (56.21%)
			Yes	402 (43.79%)
Post‐operative chemotherapy			Yes	410 (44.66%)
			No	478 (52.07%)
			NA	30 (3.27%)

**FIGURE 2:**
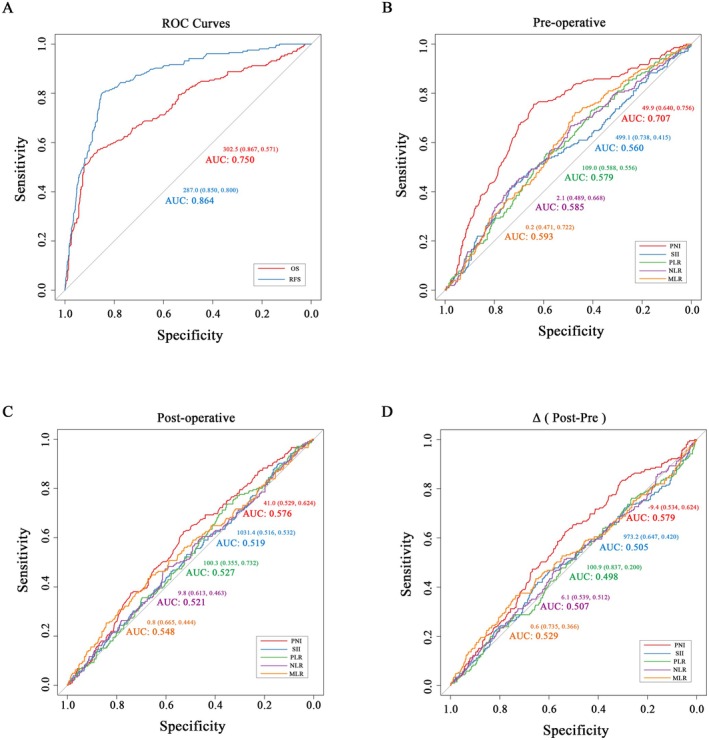
ROC curves of NIIs for NSCLC patients at different periods. (A) The ROC curves of RFS and OS in NSCLC patients. (B) The ROC curves of preoperative NIIs in NSCLC patients (*n* = 918). (C) The ROC curves of postoperative NIIs in NSCLC patients. (D) The ROC curves of differential (Δ) NIIs in NSCLC patients.

The cutoff values for NII were calculated based on the ROC curve (Figure 2 and Figure S1). Patients were subsequently divided into two groups according to these cutoff values. The time‐dependent ROC curve analysis demonstrated that RFS provided a more accurate prognostic assessment for patients with stage IB NSCLC (AUC of RFS vs. OS: 0.864 vs. 0.750). Therefore, we selected RFS as the primary outcome measure to evaluate the prognostic value of NII in this cohort. Tables 2, 3, 4, 5, 6 describe the baseline characteristics of patients grouped according to NII. Importantly, we assessed multicollinearity among preoperative, postoperative, and delta values by calculating variance inflation factors (VIFs), with all VIF values below 5, indicating the absence of significant collinearity and confirming these as independent variables.

**TABLE 2:** baseline clinical characteristics of patients stratified by PNI.

Variables		Overall	PNI								
			Pre‐operative			Post‐operative			Δ (Post‐pre)		
			Up	Low	*p*	Up	Low	*p*	UP	Low	*p*
Age	< 65	652 (71.02%)	355 (54.45%)	297 (45.55%)	0.5216	326 (50.00%)	326 (50.00%)	0.6053	323 (49.54%)	329 (50.46%)	0.9815
	≥ 65	266 (28.98%)	151 (56.77%)	115 (43.23%)		128 (48.12%)	138 (51.88%)		132 (49.62%)	134 (50.38%)	
BMI	< 18.5	50 (5.45%)	25 (50.00%)	25 (50.00%)	0.4699	21 (42.00%)	29 (58.00%)	0.5115	27 (54.00%)	23 (46.00%)	0.2600
	18.5 ~ 24	567 (61.76%)	321 (56.61%)	246 (43.39%)		280 (49.38%)	287 (50.62%)		269 (47.44%)	298 (52.56%)	
	≥ 24	301 (32.79%)	160 (53.16%)	141 (46.84%)		153 (50.83%)	148 (49.17%)		159 (52.82%)	142 (47.18%)	
Complication	No	467 (50.87%)	259 (55.46%)	208 (44.54%)	0.8328	226 (48.39%)	241 (51.61%)	0.5128	218 (46.68%)	249 (53.32%)	0.0754
	Yes	451 (49.13%)	247 (54.77%)	204 (45.23%)		228 (50.55%)	223 (49.45%)		237 (52.55%)	214 (47.45%)	
Diameter	≤ 4	774 (84.31%)	432 (55.81%)	342 (44.19%)	*0.9803*	389 (50.26%)	385 (49.74%)	0.2592	384 (49.61%)	390 (50.39%)	*0.9461*
	> 4	144 (15.69%)	74 (51.39%)	70 (48.61%)		65 (45.14%)	79 (54.86%)		71 (49.31%)	73 (50.69%)	
Differentiate	Low	442 (48.15%)	258 (58.37%)	184 (41.63%)	0.0563	203 (45.93%)	239 (54.07%)	0.0394	227 (51.36%)	215 (48.64%)	0.2950
	Medium‐High	476 (51.85%)	248 (52.10%)	228 (47.90%)		251 (52.73%)	225 (47.27%)		228 (47.90%)	248 (52.10%)	
Gender	Female	390 (42.48%)	228 (58.46%)	162 (41.54%)	0.0802	192 (49.23%)	198 (50.77%)	0.9069	190 (48.72%)	200 (51.28%)	0.6594
	Male	528 (57.52%)	278 (52.65%)	250 (47.35%)		262 (49.62%)	266 (50.38%)		265 (50.19%)	263 (49.81%)	
Site	Upper left	231 (25.16%)	133 (57.58%)	98 (42.42%)	0.2084	116 (50.22%)	115 (49.78%)	0.4290	114 (49.35%)	117 (50.65%)	0.7160
	Lower left	150 (16.34%)	84 (56.00%)	66 (44.00%)		80 (53.33%)	70 (46.67%)		78 (52.00%)	72 (48.00%)	
	Upper right	302 (32.90%)	174 (57.62%)	128 (42.38%)		154 (50.99%)	148 (49.01%)		150 (49.67%)	152 (50.33%)	
	Centre right	65 (7.08%)	35 (53.85%)	30 (46.15%)		28 (43.08%)	37 (56.92%)		27 (41.54%)	38 (58.46%)	
	Lower right	170 (18.52%)	80 (47.06%)	90 (52.94%)		76 (44.71%)	94 (55.29%)		86 (50.59%)	84 (49.41%)	
Smoking history	No	516 (56.21%)	296 (57.36%)	220 (42.64%)	0.1214	258 (50.00%)	258 (50.00%)	0.7084	255 (49.42%)	261 (50.58%)	0.9203
	Yes	402 (43.79%)	210 (52.24%)	192 (47.76%)		196 (48.76%)	206 (51.24%)		200 (49.75%)	202 (50.25%)	

**TABLE 3:** Baseline clinical characteristics of patients stratified by SII.

Variables		Overall	SII								
			Pre‐operative			Post‐operative			Δ (Post‐pre)		
			Up	Low	*p*	UP	Low	*p*	Up	Low	*p*
Age	< 65	652 (71.02%)	185 (28.37%)	467 (71.63%)	0.1922	325 (49.85%)	327 (50.15%)	0.7105	246 (37.73%)	406 (62.27%)	0.3703
	≥ 65	266 (28.98%)	87 (32.71%)	179 (67.29%)		129 (48.50%)	137 (51.50%)		92 (34.59%)	174 (65.41%)	
BMI	< 18.5	50 (5.45%)	20 (40.00%)	30 (60.00%)	0.1555	27 (54.00%)	23 (46.00%)	0.7944	18 (36.00%)	32 (64.00%)	0.8975
	18.5 ~ 24	567 (61.76%)	171 (30.16%)	396 (69.84%)		280 (49.38%)	287 (50.62%)		206 (36.33%)	361 (63.67%)	
	≥ 24	301 (32.79%)	81 (26.91%)	220 (73.09%)		147 (48.84%)	154 (51.16%)		114 (37.87%)	187 (62.13%)	
Complication	No	467 (50.87%)	130 (27.84%)	337 (72.16%)	0.2262	235 (50.32%)	232 (49.68%)	0.5934	169 (36.19%)	298 (63.81%)	0.6868
	Yes	451 (49.13%)	142 (31.49%)	309 (68.51%)		219 (48.56%)	232 (51.44%)		169 (37.47%)	282 (62.53%)	
Diameter	≤ 4	774 (84.31%)	214 (27.65%)	560 (72.35%)	0.0023	383 (49.48%)	391 (50.52%)	0.9688	287 (37.08%)	487 (62.92%)	0.7039
	> 4	144 (15.69%)	58 (40.28%)	86 (59.72%)		71 (49.31%)	73 (50.69%)		51 (35.42%)	93 (64.58%)	
Differentiate	Low	442 (48.15%)	149 (33.71%)	293 (66.29%)	0.0091	229 (51.81%)	213 (48.19%)	0.1691	170 (38.46%)	272 (61.54%)	0.3201
	Medium‐High	476 (51.85%)	123 (25.84%)	353 (74.16%)		225 (47.27%)	251 (52.73%)		168 (35.29%)	308 (64.71%)	
Gender	Female	390 (42.48%)	94 (24.10%)	296 (75.90%)	0.0016	193 (49.49%)	197 (50.51%)	0.9868	146 (37.44%)	244 (62.56%)	0.7392
	Male	528 (57.52%)	178 (33.71%)	350 (66.29%)		261 (49.43%)	267 (50.57%)		192 (36.36%)	336 (63.64%)	
Site	Upper left	231 (25.16%)	69 (29.87%)	162 (70.13%)	0.1221	127 (54.98%)	104 (45.02%)	0.0876	87 (37.66%)	144 (62.34%)	0.5196
	Lower left	150 (16.34%)	53 (35.33%)	97 (64.67%)		74 (49.33%)	76 (50.67%)		56 (37.33%)	94 (62.67%)	
	Upper right	302 (32.90%)	81 (26.82%)	221 (73.18%)		131 (43.38%)	171 (56.62%)		100 (33.11%)	202 (66.89%)	
	Centre right	65 (7.08%)	13 (20.00%)	52 (80.00%)		32 (49.23%)	33 (50.77%)		27 (41.54%)	38 (58.46%)	
	Lower right	170 (18.52%)	56 (32.94%)	114 (67.06%)		90 (52.94%)	80 (47.06%)		68 (40.00%)	102 (60.00%)	
Smoking history	No	516 (56.21%)	135 (26.16%)	381 (73.84%)	0.0092	258 (50.00%)	258 (50.00%)	0.7084	195 (37.79%)	321 (62.21%)	0.4893
	Yes	402 (43.79%)	137 (34.08%)	265 (65.92%)		196 (48.76%)	206 (51.24%)		143 (35.57%)	259 (64.43%)	

**TABLE 4:** Baseline clinical characteristics of patients stratified by PLR.

Variables		Overall	PLR								
			Pre‐operative			Post‐operative			Δ (Post‐pre)		
			Up	Low	*p*	Up	Low	*p*	Up	Low	*p*
Age	< 65	652 (71.02%)	285 (43.71%)	367 (56.29%)	0.4842	441 (67.64%)	211 (32.36%)	0.2322	114 (17.48%)	538 (82.52%)	0.6301
	≥ 65	266 (28.98%)	123 (46.24%)	143 (53.76%)		169 (63.53%)	97 (36.47%)		43 (16.17%)	223 (83.83%)	
BMI	< 18.5	50 (5.45%)	33 (66.00%)	17 (34.00%)	0.0035	34 (68.00%)	16 (32.00%)	0.2606	9 (18.00%)	41 (82.00%)	0.8942
	18.5 ~ 24	567 (61.76%)	253 (44.62%)	314 (55.38%)		387 (68.25%)	180 (31.75%)		99 (17.46%)	468 (82.54%)	
	≥ 24	301 (32.79%)	122 (40.53%)	179 (59.47%)		189 (62.79%)	112 (37.21%)		49 (16.28%)	252 (83.72%)	
Complication	No	467 (50.87%)	202 (43.25%)	265 (56.75%)	0.4604	311 (66.60%)	156 (33.40%)	0.9238	84 (17.99%)	383 (82.01%)	0.4688
	Yes	451 (49.13%)	206 (45.68%)	245 (54.32%)		299 (66.30%)	152 (33.70%)		73 (16.19%)	378 (83.81%)	
Diameter	≤ 4	774 (84.31%)	334 (43.15%)	440 (56.85%)	0.0678	515 (66.54%)	259 (33.46%)	0.8951	129 (16.67%)	645 (83.33%)	0.4163
	> 4	144 (15.69%)	74 (51.39%)	70 (48.61%)		95 (65.97%)	49 (34.03%)		28 (19.44%)	116 (80.56%)	
Differentiate	Low	442 (48.15%)	210 (47.51%)	232 (52.49%)	0.0715	286 (64.71%)	156 (35.29%)	0.2812	78 (17.65%)	364 (82.35%)	0.6728
	Medium‐High	476 (51.85%)	198 (41.60%)	278 (58.40%)		324 (68.07%)	152 (31.93%)		79 (16.60%)	397 (83.40%)	
Gender	Female	390 (42.48%)	181 (46.41%)	209 (53.59%)	0.3029	269 (68.97%)	121 (31.03%)	0.1637	62 (15.90%)	328 (84.10%)	0.4047
	Male	528 (57.52%)	227 (42.99%)	301 (57.01%)		341 (64.58%)	187 (35.42%)		95 (17.99%)	433 (82.01%)	
Site	Upper left	231 (25.16%)	108 (46.75%)	123 (53.25%)	0.1137	163 (70.56%)	68 (29.44%)	0.0283	42 (18.18%)	189 (81.82%)	0.5807
	Lower left	150 (16.34%)	68 (45.33%)	82 (54.67%)		98 (65.33%)	52 (34.67%)		26 (17.33%)	124 (82.67%)	
	Upper right	302 (32.90%)	126 (41.72%)	176 (58.28%)		181 (59.93%)	121 (40.07%)		45 (14.90%)	257 (85.10%)	
	Centre right	65 (7.08%)	21 (32.31%)	44 (67.69%)		44 (67.69%)	21 (32.31%)		15 (23.08%)	50 (76.92%)	
	Lower right	170 (18.52%)	85 (50.00%)	85 (50.00%)		124 (72.94%)	46 (27.06%)		29 (17.06%)	141 (82.94%)	
Smoking history	No	516 (56.21%)	231 (44.76%)	285 (55.23%)	0.8234	357 (69.19%)	159 (30.81%)	0.0466	82 (15.89%)	434 (84.11%)	0.2696
	Yes	402 (43.79%)	177 (44.02%)	225 (55.97%)		253 (62.94%)	149 (37.06%)		75 (18.66%)	327 (81.34%)	

## 8 The Prognostic Value of NII

**FIGURE 3:**
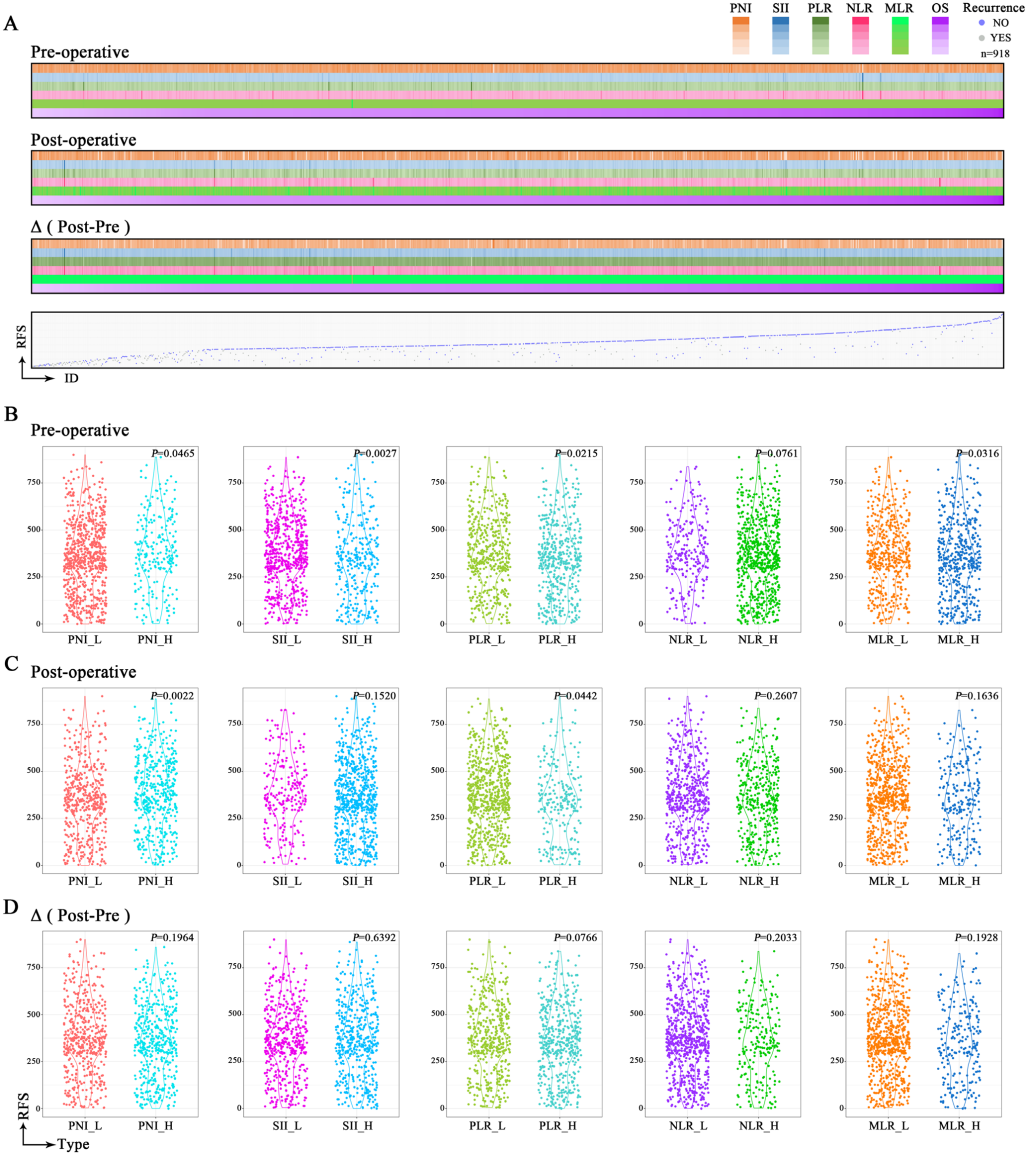
Overview of association between RFS and NIIs in NSCLC patients. (A) The characteristic heatmap mapping of blood NIIs among NSCLC patients. (B) Correlation analysis of preoperative NIIs levels with RFS in NSCLC patients. (C) Correlation analysis of postoperative NIIs levels with RFS in NSCLC patients. (D) Correlation analysis of levels of postoperative versus preoperative (Δ) NIIs differences with RFS in NSCLC patients. (*n* = 918).

Firstly, we observed a significant decrease in PNI levels in postoperative stage IB NSCLC patients, while SII, PLR, NLR, and MLR values showed a marked increase postoperatively (*p* < 0.0001) (Figure S2). Prior to assessing the prognostic value of various NII indicators for stage IB NSCLC patients, we conducted descriptive statistics on RFS across different groups of each NII at each period. The results indicated significant RFS differences between patients with high and low preoperative levels of PNI, SII, PLR, and MLR (*p* < 0.05). Patients with higher preoperative PNI or lower SII, PLR, and MLR had better RFS (Figure 3). For postoperative NII, the results indicated significant RFS differences between patients with high and low postoperative levels of PNI and PLR (*p* < 0.05). Patients with a higher postoperative PNI or lower PLR demonstrated better RFS (Figure 3). We also analyzed the relationship between pre‐ to postoperative changes in NII values and RFS. Results showed no significant RFS differences between high and low change groups (*p* > 0.05) (Figure 3). The analysis of OS was also conducted in Figure S3.

**TABLE 5:** Baseline clinical characteristics of patients stratified by NLR.

Variables		Overall	NLR								
			Pre‐operative			Post‐operative			Δ (post‐pre)		
			Up	Low	p	Up	Low	p	Up	Low	p
Age	< 65	652 (71.02%)	33 3 (51.07%)	319 (48.93%)	0.0092	252 (38.65%)	400 (61.35%)	0.1985	294 (45.09%)	358 (54.91%)	0.0293
	≥ 65	266 (28.98%)	161 (60.523%)	105 (39.47%)		115 (43.23%)	151 (56.77%)		141 (53.01%)	125 (46.99%)	
BMI	< 18.5	50 (5.45%)	29 (58.00%)	21 (42.00%)	0.6588	18 (36.00%)	32 (64.00%)	0.6461	21 (42.00%)	29 (58.00%)	0.6875
	18.5 ~ 24	567 (61.76%)	299 (52.73%)	268 (47.27%)		223 (39.33%)	344 (60.67%)		273 (48.15%)	294 (51.85%)	
	≥ 24	301 (32.79%)	166 (55.15%)	135 (44.85%)		126 (41.86%)	175 (58.14%)		141 (46.84%)	160 (53.16%)	
Complication	No	467 (50.87%)	247 (52.89%)	220 (47.11%)	0.5686	192 (41.11%)	275 (58.89%)	0.4749	224 (47.97%)	243 (52.03%)	0.7202
	Yes	451 (49.13%)	247 (54.77%)	204 (45.23%)		175 (38.80%)	276 (61.20%)		211 (46.78%)	240 (53.22%)	
Diameter	≤ 4	774 (84.31%)	406 (52.45%)	368 (47.55%)	0.0557	314 (40.57%)	460 (59.43%)	0.3973	379 (48.97%)	395 (51.03%)	0.0262
	> 4	144 (15.69%)	88 (61.11%)	56 (38.89%)		53 (36.81%)	91 (63.19%)		56 (38.89%)	88 (61.11%)	
Differentiate	Low	442 (48.15%)	259 (58.60%)	183 (41.40%)	0.0051	191 (43.21%)	251 (56.79%)	0.0539	225 (50.90%)	217 (49.10%)	0.0396
	Medium‐high	476 (51.85%)	235 (49.37%)	241 (50.63%)		176 (36.97%)	300 (63.03%)		210 (44.12%)	266 (55.88%)	
Gender	Female	390 (42.48%)	201 (51.54%)	189 (48.46%)	0.2349	142 (36.41%)	248 (63.59%)	0.0579	18 3 (46.92%)	207 (53.08%)	0.8094
	Male	528 (57.52%)	293 (55.49%)	235 (44.51%)		225 (42.61%)	303 (57.39%)		252 (47.73%)	276 (52.27%)	
Site	Upper left	231 (25.16%)	130 (56.28%)	101 (43.72%)	0.3691	100 (43.29%)	131 (56.71%)	0.7695	117 (50.65%)	114 (49.35%)	0.5933
	Lower left	150 (16.34%)	83 (55.33%)	67 (44.67%)		60 (40.00%)	90 (60.00%)		65 (43.33%)	85 (56.67%)	
	Upper right	302 (32.90%)	158 (52.32%)	144 (47.68%)		116 (38.41%)	186 (61.59%)		140 (46.36%)	162 (53.64%)	
	Centre right	65 (7.08%)	28 (43.08%)	37 (56.92%)		27 (41.54%)	38 (58.46%)		34 (52.31%)	31 (47.69%)	
	Lower right	170 (18.52%)	95 (55.88%)	75 (44.12%)		64 (37.65%)	106 (62.35%)		79 (46.47%)	91 (53.53%)	
Smoking history	No	516 (56.21%)	278 (53.88%)	238 (46.12%)	0.9652	199 (38.57%)	317 (61.43%)	0.3223	244 (47.29%)	272 (52.71%)	0.9458
	Yes	402 (43.79%)	216 (53.73%)	186 (46.27%)		168 (41.79%)	234 (58.21%)		191 (47.51%)	211 (52.49%)	

**TABLE 6:** Baseline Clinical Characteristics of Patients Stratified by MLR.

Variables		Overall	MLR								
			Pre‐operative			Post‐operative			Δ (Post‐pre)		
			Up	Low	p	Up	Low	p	Up	Low	p
Age	< 65	652 (71.02%)	326 (50.00%)	326 (50.00%)	0.0173	209 (32.06%)	443 (67.94%)	0.1631	191 (29.29%)	461 (70.71%)	0.6449
	≥ 65	266 (28.98%)	156 (58.65%)	110 (41.35%)		98 (36.84%)	168 (63.16%)		82 (30.83%)	184 (69.17%)	
BMI	< 18.5	50 (5.45%)	31 (62.00%)	19 (38.00%)	0.0582	15 (30.00%)	35 (70.00%)	0.8680	12 (24.00%)	38 (76.00%)	0.6571
	18.5 ~ 24	567 (61.76%)	281 (49.56%)	286 (50.44%)		191 (33.69%)	376 (66.31%)		171 (30.16%)	396 (69.84%)	
	≥ 24	301 (32.79%)	170 (56.48%)	131 (43.52%)		101 (33.55%)	200 (66.45%)		90 (29.90%)	211 (70.10%)	
Complication	No	467 (50.87%)	233 (49.89%)	234 (50.11%)	0.1068	163 (34.90%)	304 (65.10%)	0.3396	153 (32.76%)	314 (67.24%)	0.0414
	Yes	451 (49.13%)	249 (55.21%)	202 (44.79%)		144 (31.93%)	307 (68.07%)		120 (26.61%)	331 (73.39%)	
Diameter	≤ 4	774 (84.31%)	387 (50.00%)	387 (50.00%)	0.0004	251 (32.43%)	523 (67.57%)	0.1314	230 (29.72%)	544 (70.28%)	0.9721
	> 4	144 (15.69%)	95 (65.97%)	49 (34.03%)		56 (38.89%)	88 (61.11%)		43 (29.86%)	101 (70.14%)	
Differentiate	Low	442 (48.15%)	239 (54.07%)	203 (45.93%)	0.3596	170 (38.46%)	272 (61.54%)	0.0019	144 (32.58%)	298 (67.42%)	0.0696
	Medium‐high	476 (51.85%)	243 (51.05%)	233 (48.95%)		137 (28.78%)	339 (71.22%)		129 (27.10%)	347 (72.90%)	
Gender	Female	390 (42.48%)	181 (46.41%)	209 (53.59%)	0.0015	111 (28.46%)	279 (71.54%)	0.0060	106 (27.18%)	284 (72.82%)	0.1449
	Male	528 (57.52%)	301 (57.01%)	227 (42.99%)		196 (37.12%)	332 (62.88%)		167 (31.63%)	361 (68.37%)	
Site	Upper left	231 (25.16%)	117 (50.65%)	114 (49.35%)	0.5386	85 (36.80%)	146 (63.20%)	0.7001	77 (33.33%)	154 (66.67%)	0.3137
	Lower left	150 (16.34%)	87 (58.00%)	63 (42.00%)		45 (30.00%)	105 (70.00%)		35 (23.33%)	115 (76.67%)	
	Upper right	302 (32.90%)	152 (50.33%)	150 (49.67%)		101 (33.44%)	201 (66.56%)		93 (30.79%)	209 (69.21%)	
	Centre right	65 (7.08%)	33 (50.77%)	32 (49.23%)		20 (30.77%)	45 (69.23%)		20 (30.77%)	45 (69.23%)	
	Lower right	170 (18.52%)	93 (54.71%)	77 (45.29%)		56 (32.94%)	114 (67.06%)		48 (28.24%)	122 (71.76%)	
Smoking history	No	516 (56.21%)	272 (52.71%)	244 (47.29%)	0.8865	151 (29.26%)	365 (70.74%)	0.0024	141 (27.33%)	375 (72.67%)	0.0700
	Yes	402 (43.79%)	210 (52.24%)	192 (47.76%)		156 (38.81%)	246 (61.19%)		132 (32.84%)	270 (67.16%)	

**TABLE 7:** Tumor marker values in patients involved in the study (*n* = 120).

Items	Pre‐operative		Post‐operative		Reference range
	Mean ± SD	Min–Max	Mean ± SD	Min–Max	
CEA	19.13 ± 73.39	0.13–800.00	20.20 ± 71.00	0.62–760.75	0–5 ng/mL
CA199	29.66 ± 55.47	2.01–600.00	36.42 ± 101.98	3.01–1000.00	0.1–27 U/mL
NSE	24.16 ± 14.85	3.94–89.62	25.85 ± 29.00	2.82–200.96	0–16 ng/mL
CYFRA21‐1	7.40 ± 8.23	0.23–50.23	7.66 ± 8.49	0.31–59.91	0.1–4 ng/mL

**TABLE 8:** Clinicopathologic features of tumor marker values in patients involved in the study (*n* = 120).

Variables	Type	Value	Subtype	Number
Age	Mean ± SD	59.83 ± 8.98		
	Min–Max	36–76		
Gender			Male	80 (66.67%)
			Female	40 (33.33%)
BMI	Mean ± SD	23.02 ± 3.26		
	Min–Max	14.90–31.80		
Complication			No	63 (52.50%)
			Yes	57 (47.50%)
Diameter	Mean ± SD	2.98–1.12		
	Min–Max	0.30–5.00		
Pathological type			Adenocarcinoma	86 (71.67%)
			Squamous	34 (28.33%)
Differentiate			Low	59 (49.17%)
			Medium‐high	61 (50.83%)
Pleural invasion			Yes	76 (63.33%)
			No	12 (10.00%)
			NA	32 (26.37%)
Site			Upper‐left	34 (28.33%)
			Upper‐right	39 (32.50%)
			Lower‐left	21 (17.50%)
			Lower‐right	19 (15.83%)
			Centre‐right	7 (5.83%)
Smoking history			No	59 (49.17%)
			Yes	61 (50.83%)
Post‐operative chemotherapy			Yes	55 (45.83%)
			No	58 (48.33%)
			NA	7 (5.83%)

**FIGURE 4:**
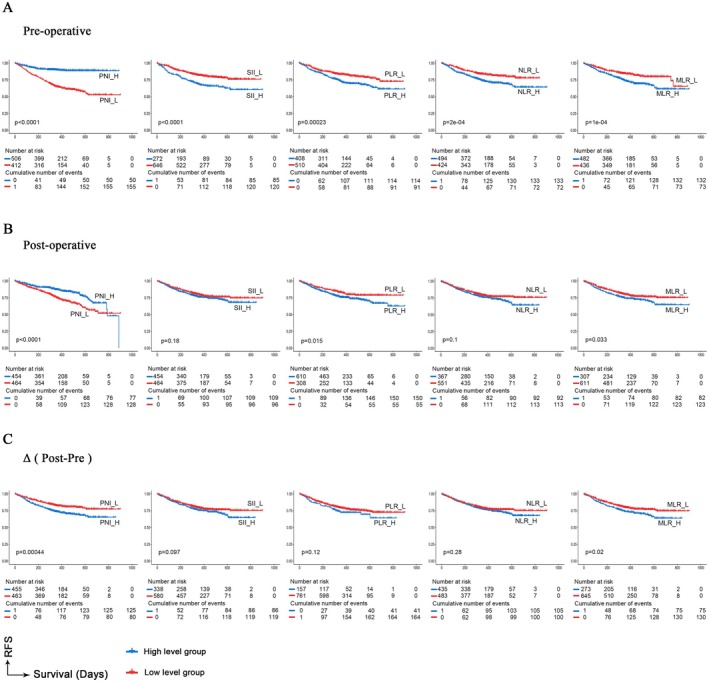
Kaplan–Meier survival curves of RFS according to NIIs at different periods.

To illustrate the relationship more vividly between independent risk factors and survival time, survival curves for 918 patients with stage IB NSCLC were generated using the Kaplan–Meier method. Specifically, patients with higher preoperative PNI levels demonstrated better RFS, whereas those with elevated preoperative levels of SII, PLR, NLR, and MLR had poorer RFS. Higher postoperative PNI levels were often associated with better RFS, while elevated SII, PLR, NLR, and MLR levels were also indicative of poorer RFS. Larger pre‐to‐postoperative differences in PNI and MLR were often associated with poorer RFS (Figure 4). The analysis of OS was also conducted in Figure S4.

**FIGURE 5:**
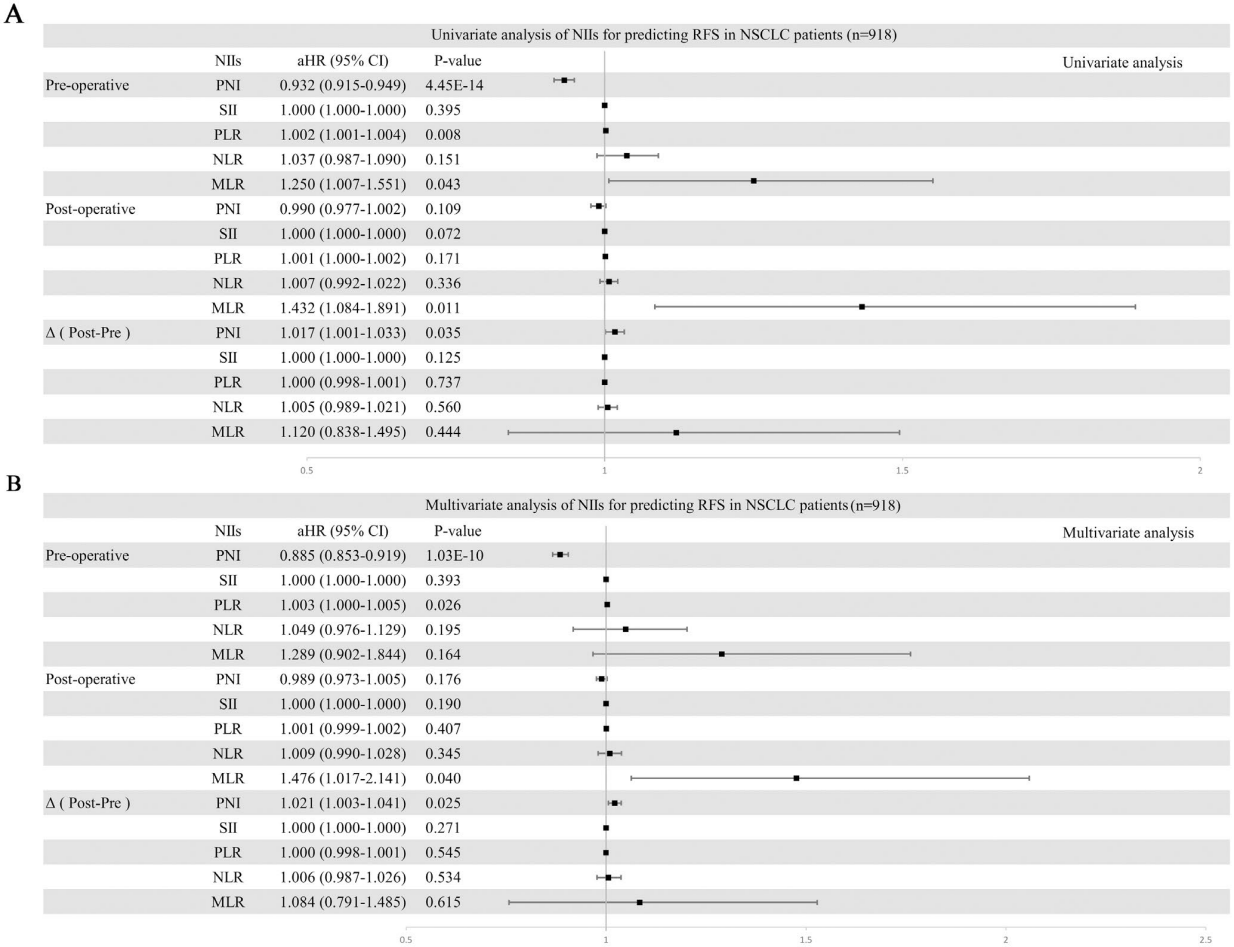
Univariate and multivariate analyses using Cox proportional hazards models using factors influencing RFS.

Univariate analysis of predictors for RFS was performed, and the results identified preoperative PNI (HR = 0.932 [0.915, 0.949], *p* < 0.0001), preoperative PLR (HR = 1.002 [1.001, 1.004], *p* = 0.008), preoperative MLR (HR = 1.250 [1.007, 1.551], *p* = 0.043), postoperative MLR (HR = 1.432 [1.084, 1.891], *p* = 0.011) and △ (post‐pre) PNI (HR = 1.017 [1.001, 1.033], *p* = 0.035) as independent prognostic factors for postoperative recurrence in stage IB NSCLC (Figure 5). Multivariate analysis of predictors for RFS was also performed, and the results identified preoperative PNI (HR = 0.885 [0.853, 0.919], *p* < 0.0001), preoperative PLR (HR = 1.003 [1.000, 1.005], *p* = 0.026), postoperative MLR (HR = 1.476 [1.017, 2.141], *p* = 0.040), and △ (post‐pre) PNI (HR = 1.021 [1.003, 1.041], *p* = 0.025) as independent prognostic factors for postoperative recurrence in stage IB NSCLC (Figure 5). The analysis of OS was also conducted in Figure S5. Considering the potential impact of postoperative adjuvant chemotherapy on the host immune‐nutritional status, we included adjuvant chemotherapy as a covariate in the Cox regression analysis of the nutritional‐immune indices (NIIs). The results showed that preoperative PNI (HR = 0.933 [0.916, 0.951], *p* < 0.0001), preoperative PLR (HR = 1.002 [1.001, 1.004], *p* = 0.008), preoperative MLR (HR = 1.268 [1.010, 1.593], *p* = 0.041), postoperative MLR (HR = 1.415 [1.065, 1.881], *p* = 0.017), and △ (post‐pre) PNI (HR = 1.018 [1.002, 1.034], *p* = 0.030) remained independent prognostic factors for postoperative recurrence and metastasis in patients with stage IB non‐small cell lung cancer (NSCLC) (Figure S6).

## 9 Preoperative PNI as the Optimal Prognostic Indicator Among NII

**FIGURE 6:**
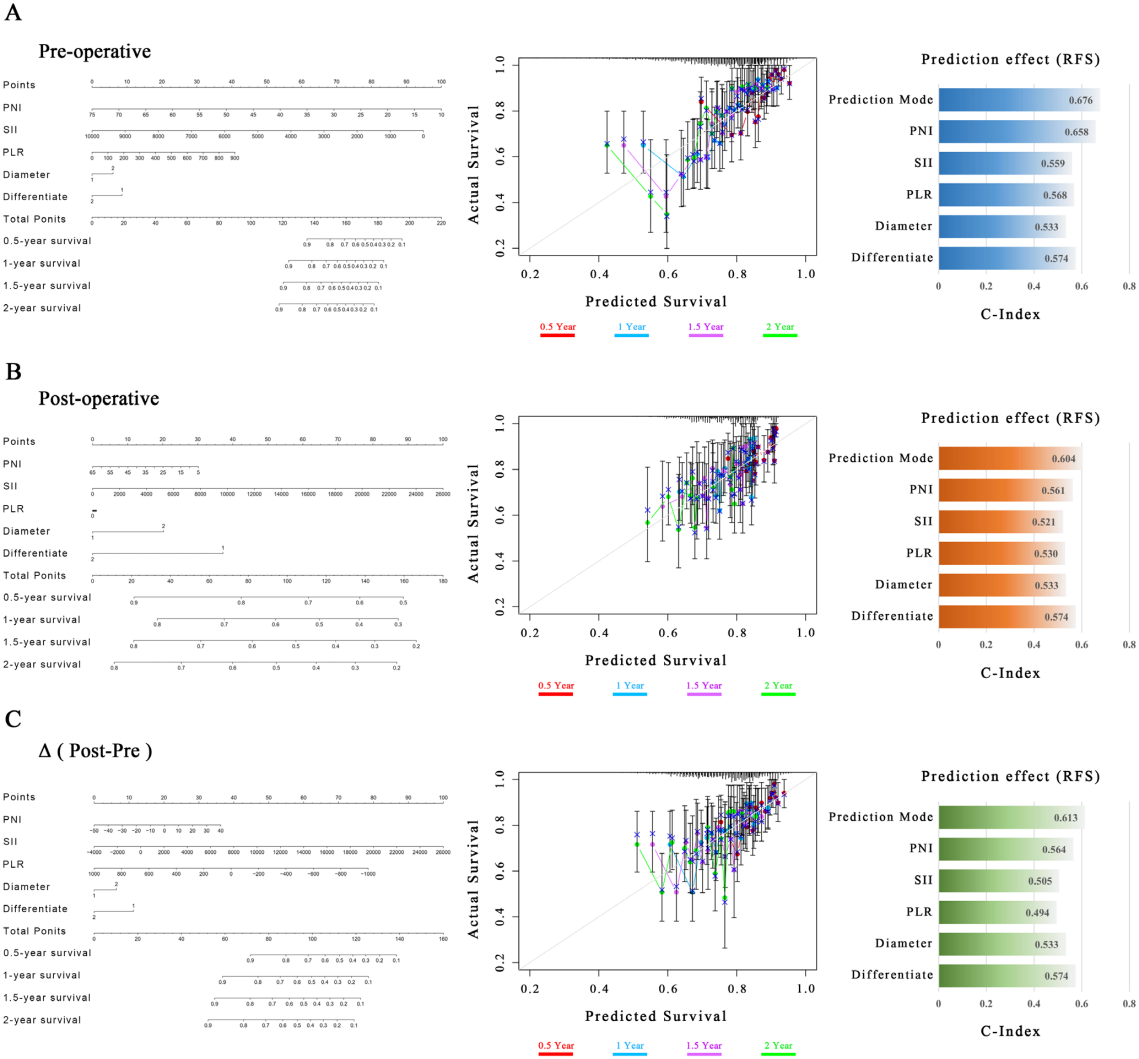
Nomogram of the developed model for predicting RFS in stage IB NSCLC patients based on the independent risk factors.

Our results have identified NII as potentially valuable prognostic markers for postoperative recurrence in stage IB NSCLC patients. To further clarify the optimal prognostic indicator within the NII, we constructed a nomogram that included all statistically significant prognostic factors in the Cox proportional hazard model, including NII indicators, age, gender, tumor size, and differentiation. The prediction results of 0.5,1,1.5, and 2‐year RFS rates were shown in Figure 6. The performance of the nomogram was assessed using the C‐index. Our results indicated that the NII system predicts RFS in stage IB NSCLC as a dynamic process, with C‐indices of 0.676, 0.604, and 0.613 for preoperative, postoperative, and pre‐to‐postoperative differences, respectively. This suggested that the preoperative NII was superior for predicting RFS. Furthermore, within the preoperative NII, we found that preoperative PNI demonstrated the best performance in predicting RFS for stage IB NSCLC, with a C‐index of 0.658, indicating that preoperative PNI may be the optimal prognostic indicator within the NII system. Additionally, the calibration curves of 0.5,1,1.5, and 2‐year RFS rates of the predicted and actual groups were shown in Figure 6, from which we could see that the calibration curves of both predicted and actual groups were close to the ideal 45° dotted line, demonstrating good agreement between predicted and actual RFS rates, affirming the reliability of the model across different time points. The analysis of OS was also conducted in Figure S7.

Additionally, time‐dependent ROC curves were generated to further evaluate the predictive accuracy of the nomogram. The AUC for preoperative PNI was 0.707, supporting its role as a robust prognostic marker in predicting the RFS of stage IB NSCLC over time (Figure S1).

## 10 Preoperative PNI Combined With Tumor Markers for Predicting RFS in Stage IB NSCLC

**FIGURE 7:**
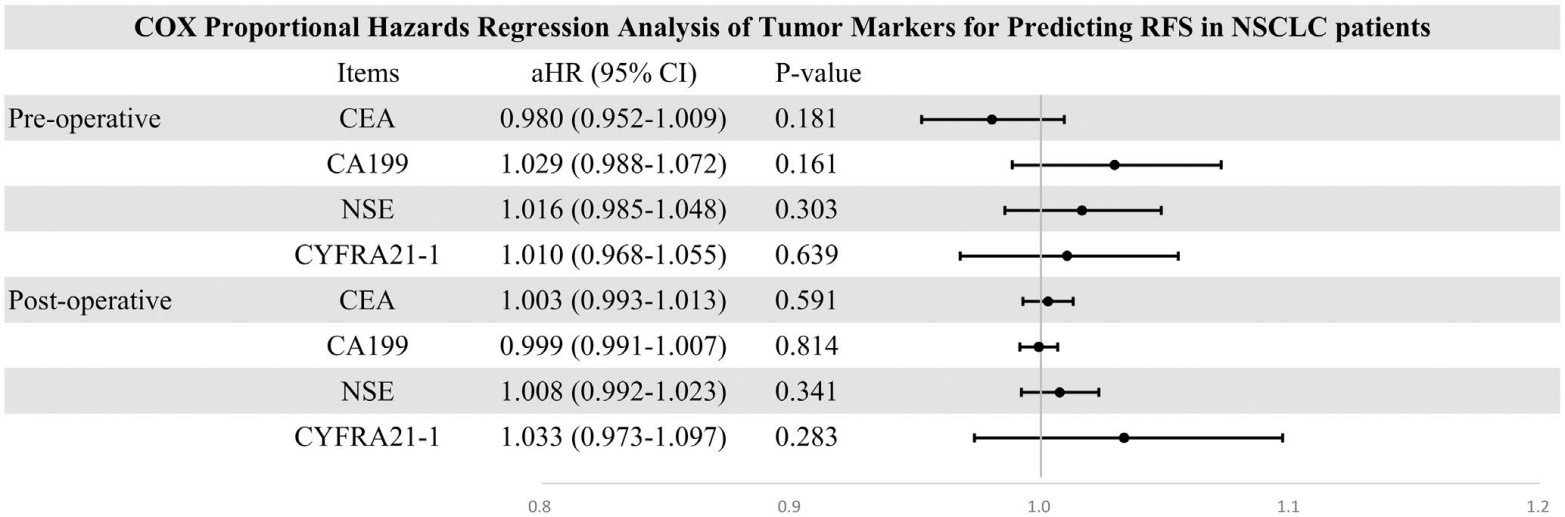
Cox proportional hazards regression analysis of tumor markers for predicting RFS in NSCLC patients.

**FIGURE 8:**
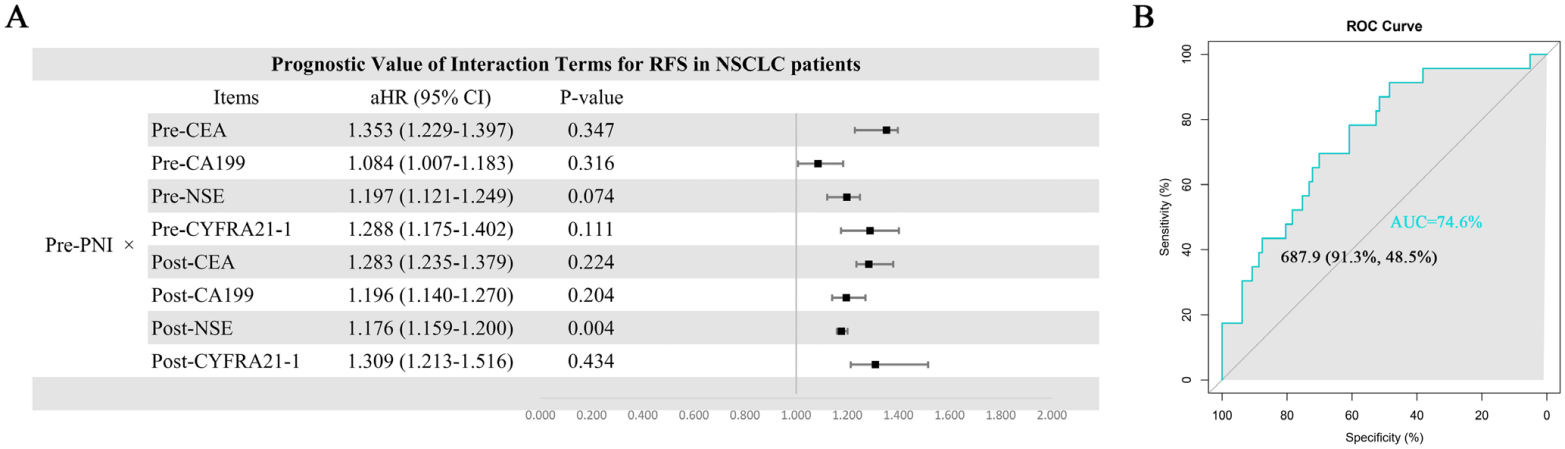
(A) Cox regression analysis of the interaction term between tumor markers and preoperative PNI predicting RFS in NSCLC patients. (B) ROC curves of the interaction terms of Pre‐PNI and Post‐NSE with RFS.

NII reflects the nutritional and immune status of patients. Stage IB NSCLC patients typically undergo routine screening for tumor markers both preoperatively and postoperatively. Therefore, the optimal prognostic indicator of NII combined with tumor markers may provide a more comprehensive prediction of RFS in stage IB NSCLC patients. We selected common tumor markers for NSCLC, including CEA, CA199, NSE, and CYFRA21‐1, to analyze their correlation with RFS in stage IB NSCLC patients. In the analysis of the correlation between tumor markers and RFS in stage IB NSCLC patients, the results revealed that neither preoperative nor postoperative tumor markers could reliably indicate the RFS of the patients (Figure 7). Subsequently, we investigated the interaction between preoperative PNI and tumor markers and assessed their association with RFS. The findings indicated that the interaction between preoperative PNI and postoperative NSE emerged as an independent prognostic factor for RFS in stage IB NSCLC patients (HR = 1.176 [95% CI: 1.159–1.200], *p* = 0.004). Furthermore, a larger interaction value of preoperative PNI and postoperative NSE was often associated with poorer RFS (Figure 8). Through receiver operating characteristic (ROC) curve analysis, we determined the optimal cutoff value of the prePNI‐postNSE interaction term for predicting RFS to be 687.9 (sensitivity = 91.3%, specificity = 48.5%, AUC = 0.746). The results suggest that during postoperative follow‐up, patients with a prePNI‐postNSE interaction term > 687.9 may have a higher risk of tumor recurrence, whereas those with an interaction term ≤ 687.9 are likely to exhibit a relatively lower recurrence risk. The analysis of OS was also conducted in Figures S8 and S9.

## 11 Discussion

To the best of our knowledge, this study was the first to systematically integrate five well‐established blood‐based nutritional and immune composite indices and evaluate their associations with postoperative recurrence and survival risks among stage IB NSCLC patients from a multi‐dimensional perioperative perspective (preoperative, postoperative, and pre‐to‐postoperative change). Our findings identified a robust NII‐based assessment strategy, with preoperative PNI emerging as a particularly strong prognostic indicator. These results offer valuable insights for recurrence risk assessment and follow‐up strategies in stage IB NSCLC patients.

The survival outcomes of stage IB NSCLC patients undergoing surgery vary significantly due to tumor heterogeneity and the absence of standardized adjuvant treatment guidelines. Postoperative recurrence remains a key factor in poor prognosis [19], highlighting the need for effective perioperative recurrence risk assessment and timely intervention to prevent recurrence and improve survival outcomes. Recent studies have sought reliable markers for assessing recurrence risk in stage IB NSCLC patients. Wang et al., utilizing TCGA data, constructed a 12‐gene prognostic model for predicting the prognosis of stage IA and IB lung squamous carcinoma from a tumor genomics perspective [20]. Similarly, Xu et al. developed a 10‐gene prognostic model (the Yin and Yang Mean Ratio (YMR) signature) using 741 tumor samples from multiple platforms, aimed at guiding postoperative adjuvant therapy [21]. Furthermore, in the realm of molecular biomarkers, high expression of proteins such as p53, CD133, phosphorylated AKT, and CUG‐binding protein (CUGBP1) has been closely linked to recurrence in stage IB NSCLC [22, 23, 24, 25], positioning them as potential indicators of recurrence risk. In addition to molecular markers, patient and tumor characteristics have also been explored. Merritt RE et al. identified histology, lymph vascular invasion (LVI), and smoking status as risk factors for recurrence [6]. Zhang et al. further found that visceral pleura invasion (VPI), micropapillary structures, tumor size, and preoperative serum levels of CEA and CYFRA21‐1 were strong predictors of recurrence, particularly in patients receiving adjuvant chemotherapy [26]. Given the established link between tumor progression, treatment response, and patients' nutritional and immune status, our study built upon these findings by focusing on a targeted cohort and incorporating multi‐variable blood indices, yielding a straightforward yet effective model for recurrence prediction to examine the relationship between perioperative recurrence events and five well‐established nutritional and immune indices (NII) across perioperative stages in stage IB NSCLC. Our findings identified preoperative PNI as the optimal indicator for recurrence risk, offering a novel approach to risk assessment and adjuvant treatment strategies in postoperative stage IB NSCLC patients.

In clinical practice, there is a growing need for cost‐effective and accessible biomarkers to evaluate postoperative outcomes, including survival, recurrence, and metastasis. These biomarkers are instrumental in aiding timely therapeutic decision‐making, ultimately supporting individualized management and improved prognosis. Blood‐based nutritional and immune indicators, including the prognostic nutritional index (PNI), systemic immune‐inflammation index (SII), neutrophil‐to‐lymphocyte ratio (NLR), platelet‐to‐lymphocyte ratio (PLR), and monocyte‐to‐lymphocyte ratio (MLR), show promising clinical utility as predictive markers for recurrence risk following treatment. Emerging studies indicated that a patient's nutritional and immune status could modulate tumor progression, metastasis, and treatment response by regulating inflammatory responses within the tumor microenvironment [12]. PNI, SII, NLR, PLR, and MLR have proven to be highly sensitive and specific prognostic markers across various cancer types [13, 14, 15, 16, 17]. Additionally, as these indices are based on routine laboratory data, they are both reliable and easily accessible. However, the prognostic value of these blood‐based nutritional and immune indices (NII) for postoperative recurrence in stage IB NSCLC remains under‐explored. Treatment modalities also impact individual nutritional, inflammatory, and immune statuses, raising the question of which perioperative period (preoperative, postoperative, or pre‐post difference) NII best reflects recurrence risk. To address this, our study developed a 15‐variable NII‐based assessment system encompassing preoperative, postoperative, and change (post‐pre) values of PNI, SII, NLR, PLR, and MLR, using recurrence‐free survival (RFS) as the outcome measure. Through univariate and multivariate Cox regression analyses, we identified preoperative PNI, postoperative MLR, and △ (post‐pre) PNI as independent prognostic factors for postoperative recurrence in stage IB NSCLC patients. We further internally evaluated these findings by constructing a nomogram model, stratifying patients into risk groups based on age, gender, tumor size, and differentiation, to elucidate the prognostic relevance of these selected NII indicators across different risk subgroups. Our findings indicated that preoperative PNI served as the most robust predictor of postoperative recurrence in IB NSCLC patients. Our results align with those of Shoji F et al., who conducted a retrospective analysis of 141 surgically treated stage I NSCLC patients, finding a strong association between preoperative PNI and recurrence [27]. Our study built upon this with a larger, more targeted cohort of stage IB patients, and by applying a quantifiable, multi‐variable nomogram model, we further validate preoperative PNI as a reliable recurrence predictor. A meta‐analysis by Li et al. systematically evaluated the prognostic value of preoperative PNI in lung cancer, concluding that ‘PNI was an independent prognostic indicator for lung cancer and can serve as a novel biomarker to help guide clinical practice and improve clinical outcomes of lung cancer patients’—findings that are consistent with our results. Notably, their study included patients with stage I–IV NSCLC, demonstrating PNI's robust prognostic value across the entire lung cancer population [28]. Furthermore, research by Yan et al. has shown that PNI also serves as a reliable predictive marker for advanced lung cancer patients receiving immune checkpoint inhibitor (ICI) therapy [29], further validating the reliability of PNI as a prognostic indicator in lung cancer patients.

Studies have demonstrated that blood‐based tumor marker (TM) levels are closely linked to tumor growth, progression, metastasis, and survival following treatment, highlighting their potential in cancer diagnosis, treatment monitoring, and prognosis [30, 31, 32]. In routine postoperative follow‐ups for lung cancer patients, clinicians frequently request tumor marker testing. However, our clinical experience and study findings indicate that tumor markers alone often fall short in providing sufficient prognostic insight, especially for stage IB lung cancer patients. To enhance prognostic accuracy, we took an innovative approach by combining preoperative PNI—identified as the most effective marker among nutritional and immune indices—with traditional tumor markers (CEA, CA19‐9, NSE, and CYFRA21‐1) in predicting postoperative recurrence for IB‐stage NSCLC. We aimed to enhance the sensitivity and accuracy of recurrence prediction through this combined system, providing practical guidance for long‐term follow‐up. Interestingly, while individual TMs did not significantly correlate with recurrence, combining them within a pre‐PNI‐TM system markedly improved their predictive relevance, with postoperative NSE showing a significant association with recurrence when combined with pre‐PNI. Our study identified that the interaction term between Pre‐PNI and postoperative NSE serves as an independent prognostic factor for postoperative recurrence/metastasis in stage IB NSCLC (HR = 1.176 [95% CI: 1.159–1.200], *p* = 0.004). Specifically, each unit increase in the pre‐PNI×postoperative NSE interaction term—calculated as (10 × serum albumin [g/dL] + 5 × total lymphocyte count [/nL] × NSE)‐corresponds to a 1.176‐fold increased risk of postoperative recurrence in stage IB NSCLC patients. This suggested that combining tumor marker levels with preoperative PNI data enables clinicians to enhance the accuracy of recurrence risk evaluation during postoperative follow‐up in stage IB patients. Neuron‐specific enolase (NSE) is an isoenzyme of enolase involved in glycolysis, primarily expressed in neurons and neuroendocrine cells. NSE is commonly used as a serum tumor marker for small cell lung cancer (SCLC) and neuroendocrine tumors, aiding in diagnosis and treatment monitoring. However, recent studies have shown that NSE levels can also be elevated in patients with non‐small cell lung cancer (NSCLC), particularly in subgroups characterized by neuroendocrine differentiation or aggressive biological behavior, drawing increasing clinical attention. Multiple studies have confirmed that elevated serum NSE levels are closely associated with poor prognosis in NSCLC patients. For example, Yu et al. found in a cohort of 481 NSCLC patients that high levels of preoperative serum NSE were correlated with worse overall survival in operable NSCLC patients. The study concluded that NSE could serve as an independent prognostic marker and provide valuable support for clinical risk stratification [33]. In addition, Yan et al. reported that elevated NSE was significantly associated with postoperative recurrence, distant metastasis, and shorter disease‐free survival in NSCLC, suggesting its potential utility in monitoring disease progression [34]. Nevertheless, the prognostic value of NSE in NSCLC remains controversial. In our study, we found that NSE alone was not directly correlated with postoperative recurrence or metastasis in stage IB NSCLC. However, the interaction between NSE and preoperative perineural invasion (Pre‐PNI) could serve as a significant prognostic indicator, highlighting the potential of combining biomarkers to improve prognostic accuracy. Our team will further validate these findings in large‐scale retrospective and prospective studies to confirm the feasibility and efficacy of using pre‐PNI combined with TM results for postoperative recurrence monitoring in IB NSCLC patients.

Despite the potential of the NII‐based assessment strategy, this study has limitations. First, variability in follow‐up duration may introduce bias affecting result accuracy. Second, recent research suggests that genetic mutations (e.g., EGFR, ALK, ROS‐1) play critical roles in recurrence and metastasis, but due to limited availability of gene mutation testing methods (NGS, IHC, FISH) during our study period (2005–2014), we did not adjust for mutation‐related effects, which could affect the reliability of our NII‐based recurrence predictions. Third, the study's modest sample size of 120 patients with comprehensive preoperative and postoperative TM data limited the statistical power of the pre‐PNI and TM interactions. Future studies will incorporate a larger patient pool and more follow‐up time points to validate the predictive reliability of our NII‐based assessment strategy. Lastly, as our patients were exclusively from a single center (West China Hospital), further validation using authoritative public lung cancer databases with blood‐based nutritional and immune data will be essential to confirm our findings.

## 12 Conclusion

Our study demonstrated that a prognostic assessment strategy based on Nutritional and Immune Indices (NII) is a valuable tool for predicting postoperative recurrence in stage IB NSCLC patients. Within the NII framework, preoperative PNI emerged as the most effective predictor of recurrence. Additionally, combining preoperative PNI with tumor markers could provide a promising and reliable indicator for assessing postoperative recurrence risk in this patient group.

## Author Contributions

**Jianqi Hao:** conceptualization, validation, writing – review and editing, writing – original draft, methodology, supervision. **Song He:** software, data curation, writing – review and editing, writing – original draft, formal analysis. **Cong Chen:** data curation, formal analysis, investigation, writing – original draft, writing – review and editing. **Wenying Xu:** data curation, investigation, writing – original draft. **Xiaohu Hao:** data curation, investigation, writing – original draft. **Nanzhi Luo:** formal analysis, investigation, writing – original draft. **Chenglin Guo:** investigation, writing – original draft, writing – review and editing. **Qiang Pu:** supervision, writing – review and editing. **Lunxu Liu:** conceptualization, writing – review and editing, funding acquisition, supervision.

## Ethics Statement

This study received approval from the Institutional Review Board of West China Hospital (No. 2025639). This project was registered in the Chinese Clinical Trial Registry (ChiCTR2500101059) https://www.chictr.org.cn/hvshowproject.html?id=275036&v=1.0.

## Conflicts of Interest

The authors declare no conflicts of interest.

## Supporting information


**Figure S1.** ROC curves of NIIs for NSCLC patients at different periods.

**Figure S2.** Dynamic changes in Nutritional and Immune Indices (NIIs) between preoperative and postoperative periods.

**Figure S3.** Overview of association between OS and NIIs in NSCLC patients from a multi‐dimensional perioperative perspective.

**Figure S4.** Kaplan–Meier survival curves of OS according NIIs at different periods.

**Figure S5.** Univariate and multivariate analyses using Cox proportional hazards models using factors influencing OS.

**Figure S6.** Cox regression analysis of RFS stratified by NIls and covariates (*n* = 918).

**Figure S7.** Nomogram of the developed model for predicting OS in stage IB NSCLC patients based on the independent risk factors.

**Figure S8.** Cox proportional hazards regression analysis of tumor markers for predicting OS in NSCLC patients.

**Figure S9.** (A) Cox regression analysis of interaction term between tumor markers and preoperative PNI predicting OS in NSCLC patients. (B) ROC curves of interaction terms of Pre‐PNI and Post‐NSE with OS.

## Data Availability

All relevant data are within the paper. The raw data are available from the corresponding author on reasonable request. E‐mail: lunxu_liu@aliyun.com
