# Supplementary figures and images for: The Prognostic Value of Nutritional and Immune Indices for Stage IB Non‐Small Cell Lung Cancer Patients: Insights From a Retrospective Cohort Study
Source: Cancer Med. 2025 Jul 28;14(15):e71089. doi: 10.1002/cam4.71089 (PMC12301860; doi:10.1002/cam4.71089)

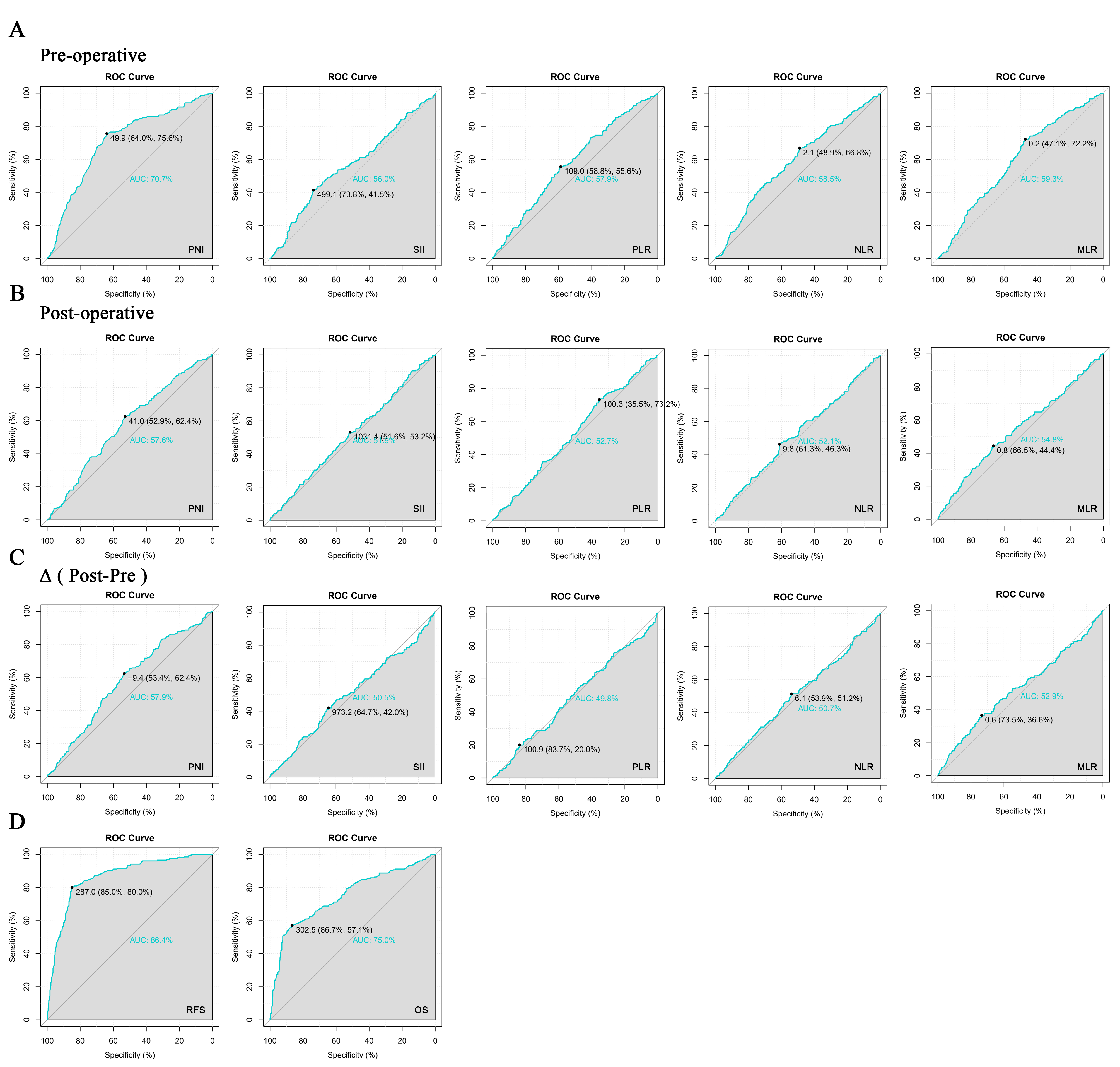

Supplement: Supplementary file 1 — Figure S1. ROC curves of NIIs for NSCLC patients at different periods. [file CAM4-14-e71089-s007.tif]

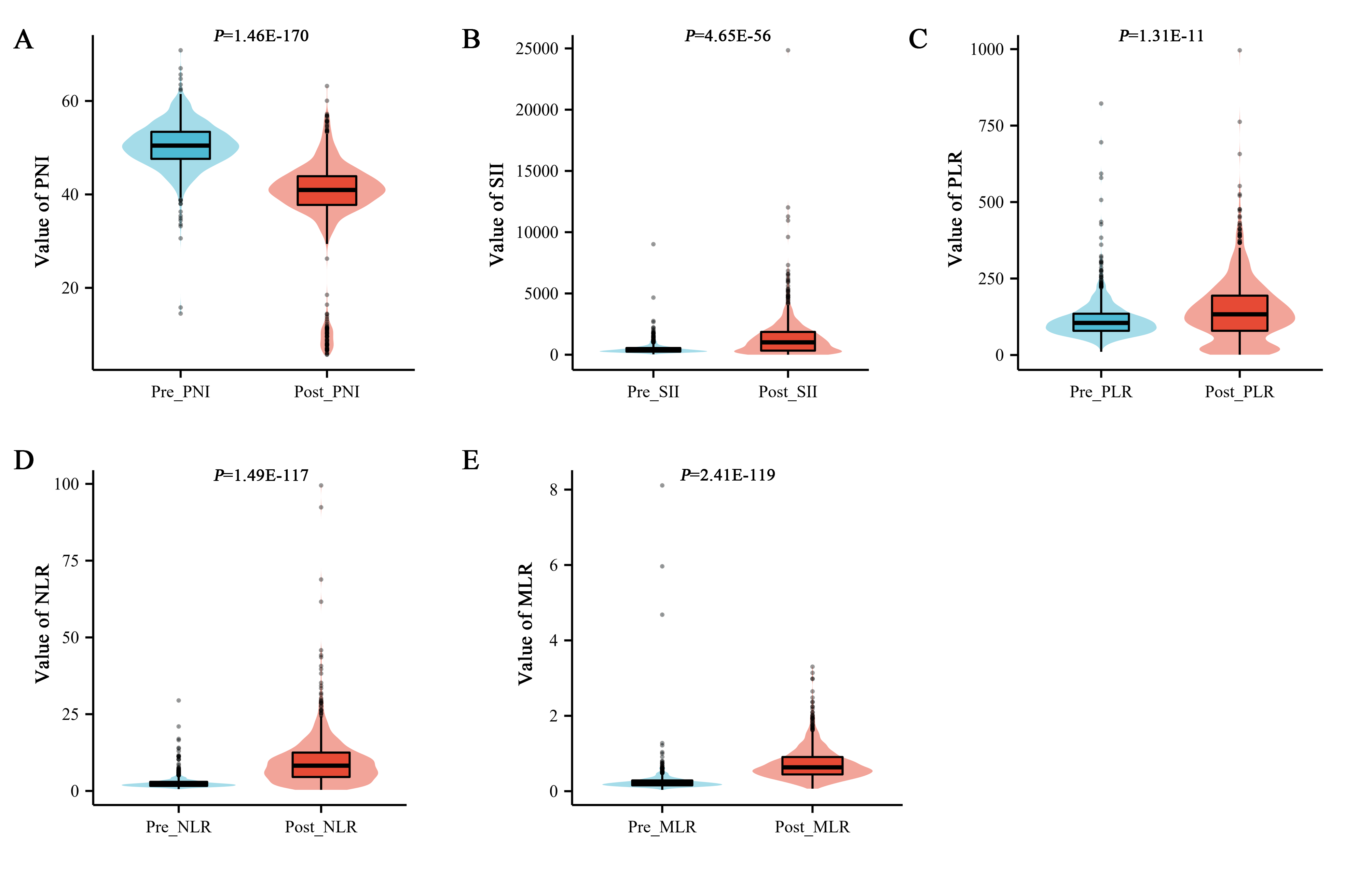

Supplement: Supplementary file 2 — Figure S2. Dynamic changes in Nutritional and Immune Indices (NIIs) between preoperative and postoperative periods. [file CAM4-14-e71089-s006.tif]

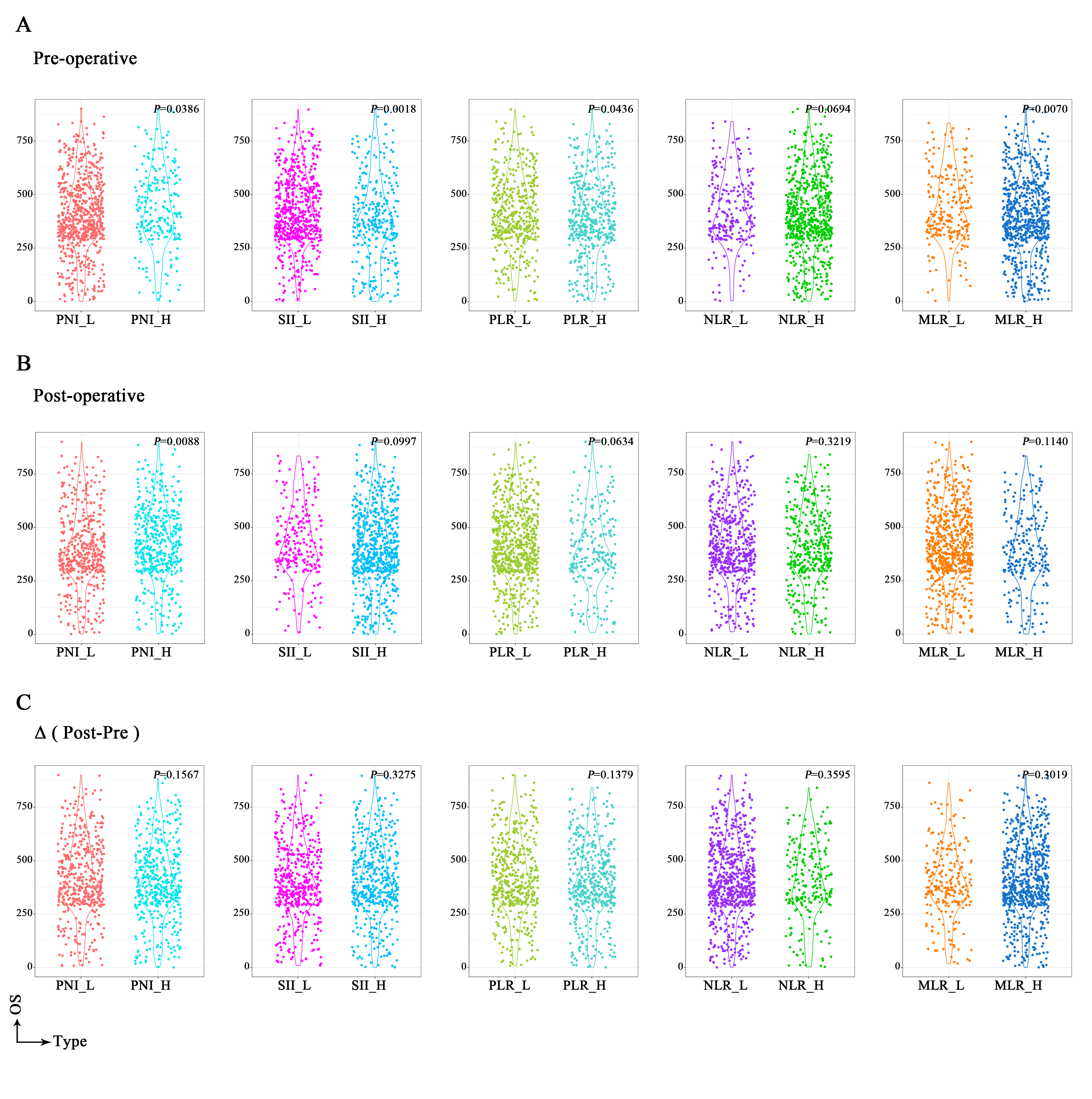

Supplement: Supplementary file 3 — Figure S3. Overview of association between OS and NIIs in NSCLC patients from a multi‐dimensional perioperative perspective. [file CAM4-14-e71089-s004.tif]

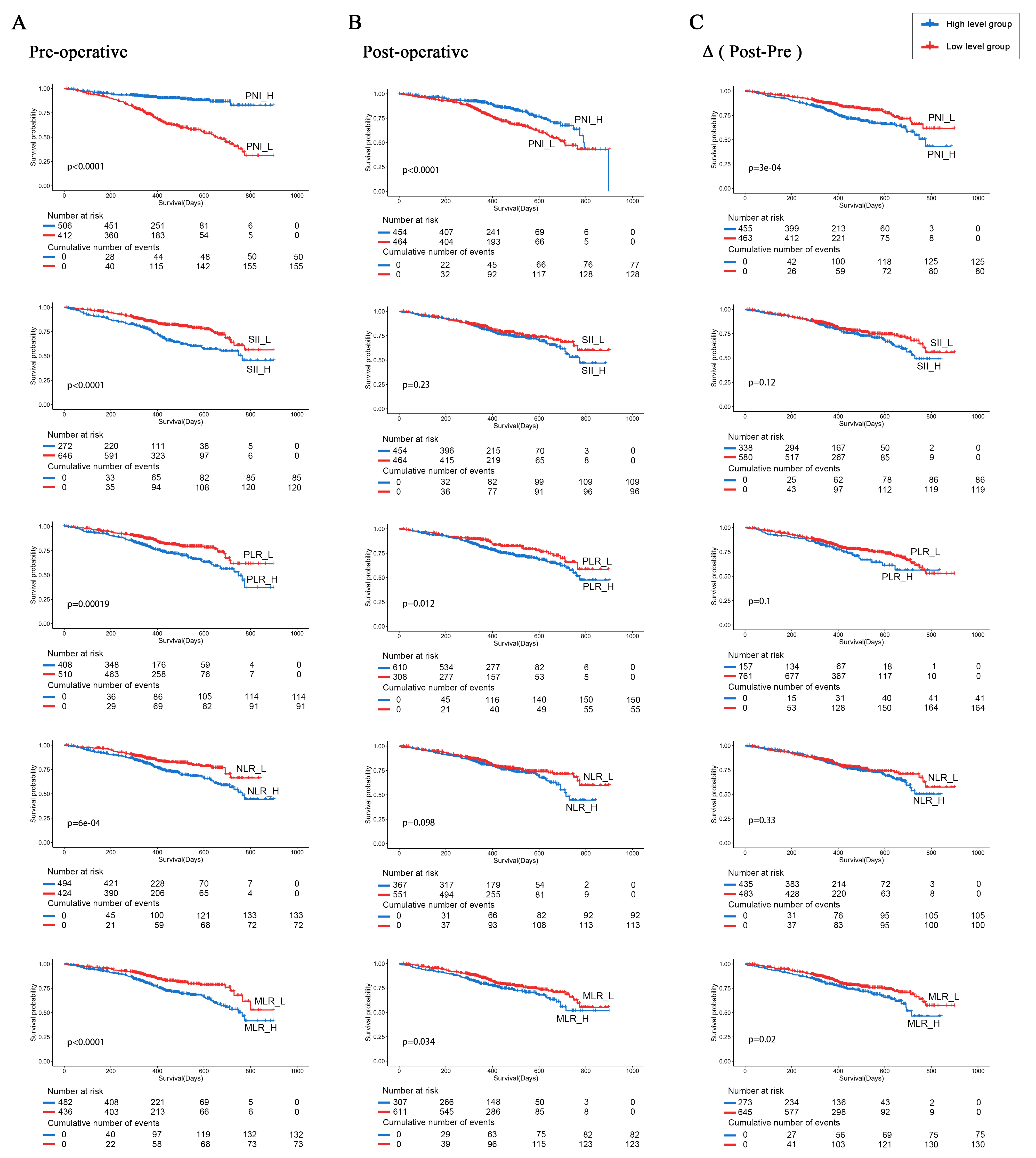

Supplement: Supplementary file 4 — Figure S4. Kaplan–Meier survival curves of OS according NIIs at different periods. [file CAM4-14-e71089-s005.tif]

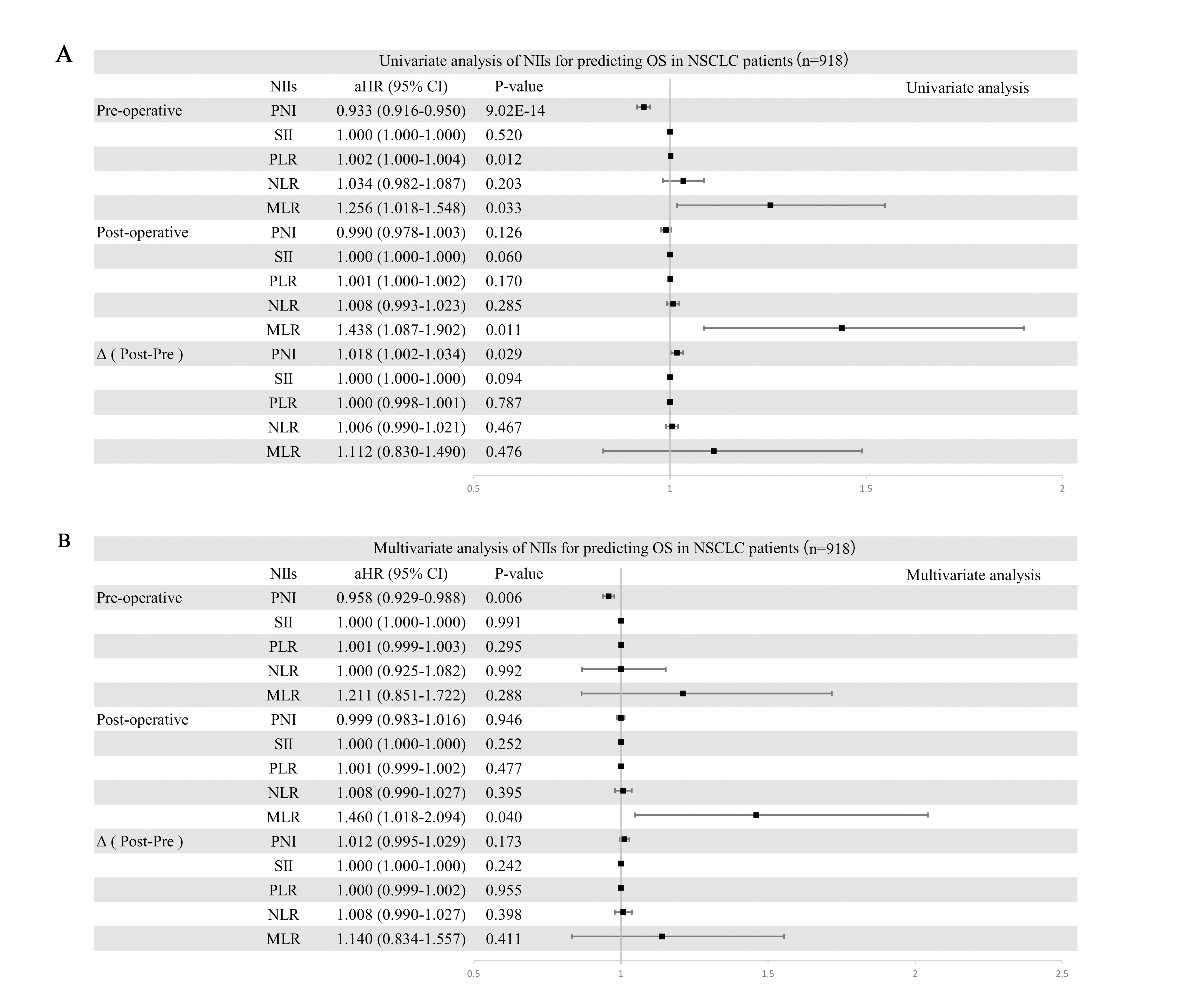

Supplement: Supplementary file 5 — Figure S5. Univariate and multivariate analyses using Cox proportional hazards models using factors influencing OS. [file CAM4-14-e71089-s001.tif]

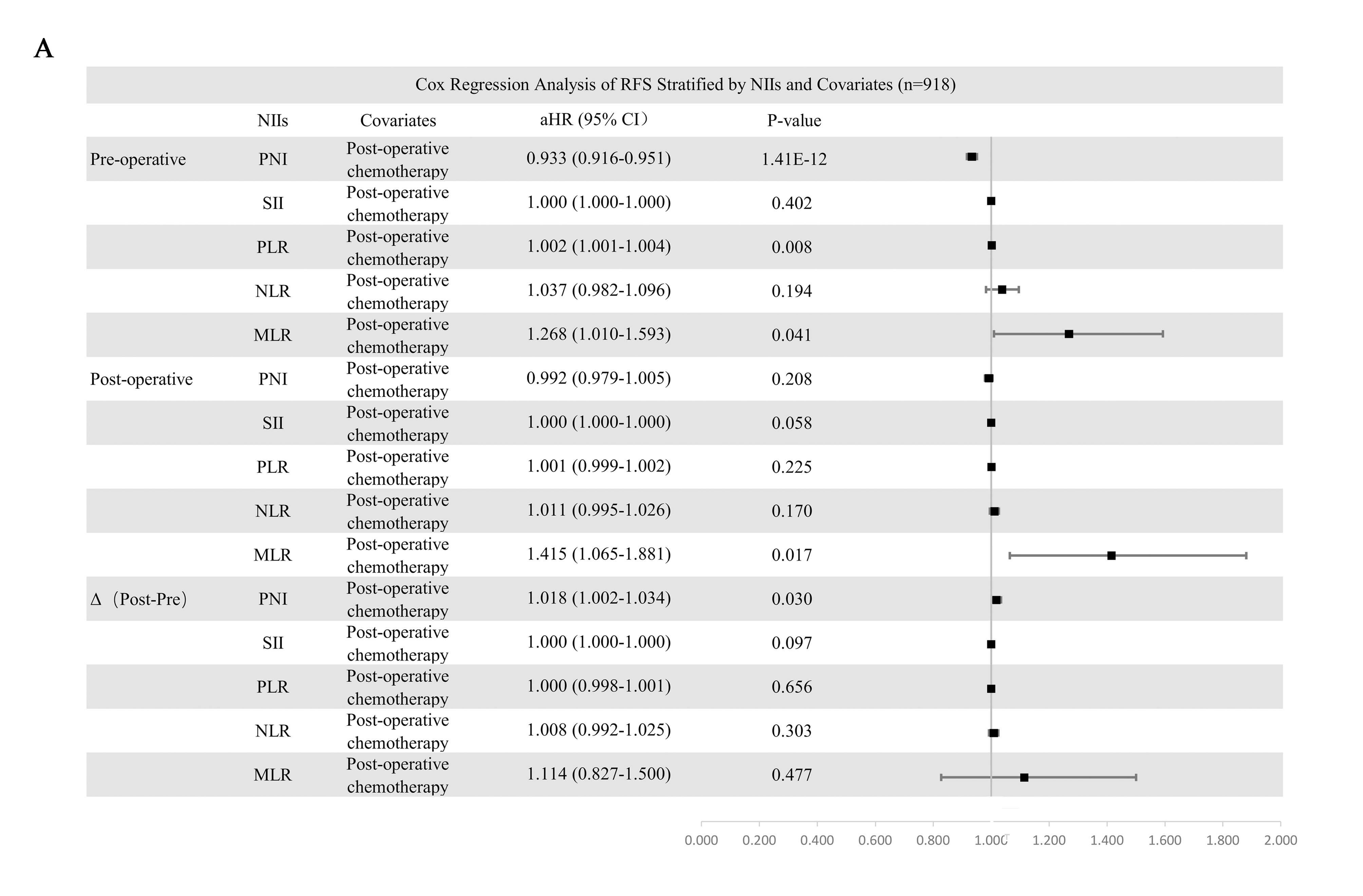

Supplement: Supplementary file 6 — Figure S6. Cox regression analysis of RFS stratified by NIls and covariates (n = 918). [file CAM4-14-e71089-s002.tif]

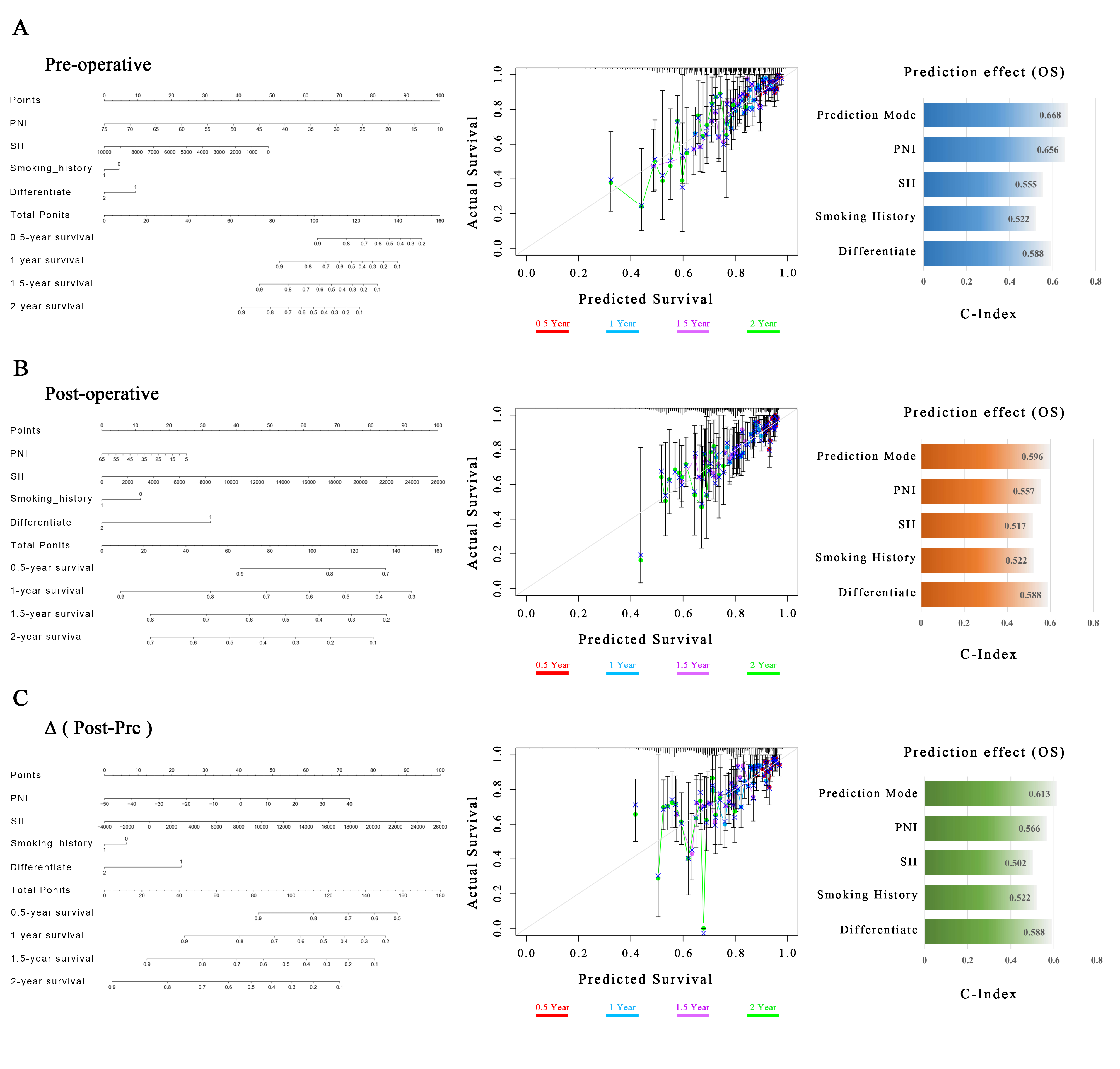

Supplement: Supplementary file 7 — Figure S7. Nomogram of the developed model for predicting OS in stage IB NSCLC patients based on the independent risk factors. [file CAM4-14-e71089-s009.tif]

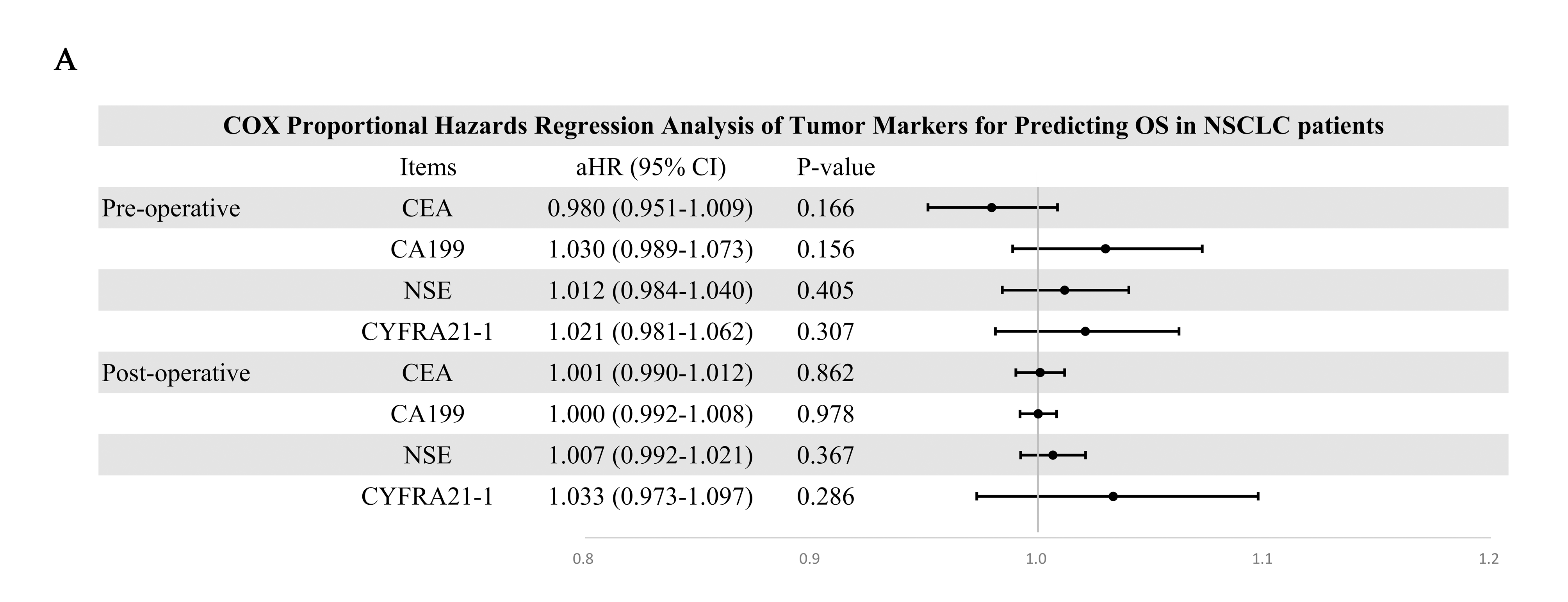

Supplement: Supplementary file 8 — Figure S8. Cox proportional hazards regression analysis of tumor markers for predicting OS in NSCLC patients. [file CAM4-14-e71089-s008.tif]

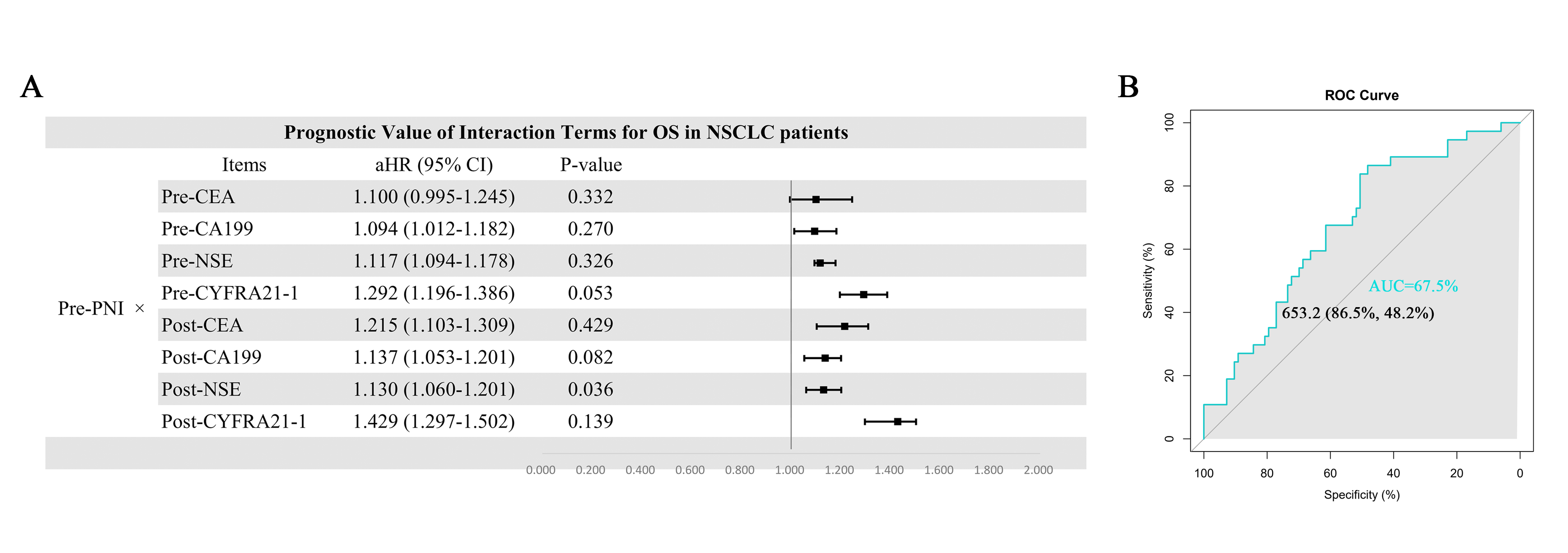

Supplement: Supplementary file 9 — Figure S9. (A) Cox regression analysis of interaction term between tumor markers and preoperative PNI predicting OS in NSCLC patients. (B) ROC curves of interaction terms of Pre‐PNI and Post‐NSE with OS. [file CAM4-14-e71089-s003.tif]
